# Chromosome and Molecular Analyses Reveal Significant Karyotype Diversity and Provide New Evidence on the Origin of *Aegilops columnaris*

**DOI:** 10.3390/plants10050956

**Published:** 2021-05-11

**Authors:** Ekaterina D. Badaeva, Nadezhda N. Chikida, Andrey N. Fisenko, Sergei A. Surzhikov, Maria K. Belousova, Hakan Özkan, Alexandra Y. Dragovich, Elena Z. Kochieva

**Affiliations:** 1 N. I. Vavilov Institute of General Genetics, Russian Academy of Sciences, Gubkina Street 3, GSP–1, 119991 Moscow, Russia; fisenko800@mail.ru (A.N.F.); dragovich@vigg.ru (A.Y.D.); 2 Engelhardt Institute of Molecular Biology, Russian Academy of Sciences, Vavilova Street 34, GSP–1, 119991 Moscow, Russia; ssergey77@mail.ru; 3 Federal Research Center, N. I. Vavilov All-Russian Institute of Plant Genetic Resources, Bolshaya Morskaya Street 44, 190121 St. Petersburg, Russia; n.chikida@mail.ru (N.N.C.); m.h.belousova@mail.ru (M.K.B.); 4 Department of Field Crops, Faculty of Agriculture, University of Çukurova, 01330 Adana, Turkey; hozkan@cu.edu.tr; 5 Federal Research Center “Fundamentals of Biotechnology”, Russian Academy of Sciences, 60 let Oktjabrya Prospect 7, Build. 1, 117312 Moscow, Russia; ekochieva@yandex.ru

**Keywords:** *Aegilops columnaris*, *Ae. neglecta*, C-banding, FISH, gliadin electrophoresis, sequencing, spacer regions of the chloroplast DNA, plastogroups, evolution

## Abstract

*Aegilops columnaris* Zhuk. is tetraploid grass species (2n = 4x = 28, U^c^U^c^X^c^X^c^) closely related to *Ae. neglecta* and growing in Western Asia and a western part of the Fertile Crescent. Genetic diversity of *Ae. columnaris* was assessed using C-banding, FISH, nuclear and chloroplast (cp) DNA analyses, and gliadin electrophoresis. Cytogenetically *Ae. columnaris* was subdivided into two groups, C-I and C-II, showing different karyotype structure, C-banding, and FISH patterns. C-I group was more similar to *Ae. neglecta*. All types of markers revealed significant heterogeneity in C-II group, although group C-I was also polymorphic. Two chromosomal groups were consistent with plastogroups identified in a current study based on sequencing of three chloroplast intergenic spacer regions. The similarity of group C-I of *Ae. columnaris* with *Ae. neglecta* and their distinctness from C-II indicate that divergence of the C-I group was associated with minor genome modifications. Group C-II could emerge from C-I relatively recently, probably due to introgression from another *Aegilops* species followed by a reorganization of the parental genomes. Most C-II accessions were collected from a very narrow geographic region, and they might originate from a common ancestor. We suggest that the C-II group is at the initial stage of species divergence and undergoing an extensive speciation process.

## 1. Introduction

*Aegilops columnaris* Zhuk. is annual tetraploid (2n = 4x = 28) grass species naturally growing in Western Asia, mainly in Turkey, Armenia, and in a western part of the Fertile Crescent [1,2,3]. It is also native to Crete, Iraq, Lebanon, Azerbaijan, and Iran but found as adventive species in France, near Marseille [1]. Despite a relatively broad distribution area, *Ae. columnaris* is uncommon throughout its range. Biodiversity Collecting Mission Database included 816 *Ae. columnaris* site records (https://www.gbif.org/), and according to Genesys, 763 accessions are currently maintained in gene banks worldwide (https://www.genesys-pgr.org/). This number, however, may be overestimated owing to a large number of potentially duplicated and incorrectly classified accessions. From the other hand, many new sites were recently discovered during collection missions. However, the new samples (e.g., reported in [4] or materials analyzed in a current study) were not included in these databases.

*Ae. columnaris* was first collected on the Ghalat plateau close to Ankara and on the slopes of Dizgurt-Dagh mountains, Turkey, by the Russian botanist P.M. Zhukovsky during expeditions of 1925–1927 to Asia Minor [5]. Since then, this species was found in other locations, mainly in Turkey, Syria, and Transcaucasia, but also in Lebanon, Iraq, Iran, and Aegean Greece (Rodos, Crete) [1,6,7]. *Ae. columnaris* grows in dry fields, roads, and hillsides [1,5], mainly on limestones, rarer on basalts on wetter environments than most *Aegilops* L. species [1,2]. In most locations, *Ae. columnaris* is found together with other *Aegilops* species, often in a mix with *Ae. neglecta* Req. ex Bertol., *Ae. biuncialis* Vis., *Ae. peregrina* (Hack. in J. Fraser) Maire and Weiller, or *Ae. triuncialis* L. [8].

*Ae. columnaris* is known to be closely related to tetraploid *Ae. neglecta* [9,10,11,12,13,14,15], but the origin of these two species was a subject of long debates and is still not clear. Analysis of meiotic chromosome pairing in intraspecific hybrids [15,16,17,18], comparison of karyotype structure [19] and C-banding patterns [13,20], molecular analysis on nuclear [11,12] and cytoplasmic DNAs [21,22] showed that one of the *Ae. columnaris* and *Ae. neglecta* genomes was contributed by the diploid species *Ae. umbellulata* Zhuk. (2n = 2x = 14, UU). Comparative sequence analysis of the nuclear U-genome specific U31 fragment in 48 accessions of each *Ae. columnaris* and *Ae. neglecta* in comparison with 72 accessions of *Ae. umbellulata* allowed to suggest that the U-genomes of *Ae. columnaris* and *Ae. neglecta* may have multiple origins [23]. Cytoplasmic genomes of *Ae. columnaris* (U^2^) and *Ae. neglecta* (U) are also similar to the cytoplasmic genome of *Ae. umbellulata* (U), indicating that *Ae. umbellulata* was the maternal parent of these tetraploid species [22,24].

The source of the second genome of *Ae. columnaris* and *Ae. neglecta* is still unknown. H. Kihara [17] suggested that it could be related to the M-genome of *Ae. comosa* Sm. in Sibth. and Sm. based on morphological comparisons and analysis of meiotic chromosome pairing in *Ae. columnaris* x *Ae. biuncialis* (2n = 4x = 28, UUMM) hybrids. He designated this genome as “modified M,” and this symbol is still used in most taxonomical systems [1,2,10,16,17,19,25]. However, the F_1_ hybrids of *Ae. columnaris* x *Ae. comosa* exhibited low chromosome pairing [15]. Differences in the patterns of variation of the repetitive nucleotide sequences [11,26], RAPD-spectra [9], the results of DArTseq-based analysis [12], comparison of karyotype structures [19], C-banding, and Fluorescence in situ hybridization (FISH)-patterns [13,20] contradicted this hypothesis. Taking into consideration the distinctness of *Ae. columnaris* genomes Dvořák [26] suggested to change its genome formula from the UM to UX^1^. More recent data of DArTseq-based analysis revealed higher similarity of the second genome of *Ae. columnaris* and *Ae. neglecta* with the genome of *Ae. speltoides* Tausch or *Ae. mutica* Boiss. [12], therefore, a new genomic formula, UT^s^, was proposed for these tetraploid species.

In a previous publication [13], we uncovered the significant karyotype diversity of *Ae. columnaris*, which was expressed in a variation of the C-banding patterns and, despite a small number of accessions studied, translocation polymorphism. In this paper, however, the translocations were classified tentatively due to the lack of standard genetic nomenclature of *Ae. columnaris* chromosomes. The problem of chromosome classification was solved later when a set of wheat-*Ae. columnaris* (K−1193) introgression lines was developed and cytogenetically characterized [20].

These introgression lines also enabled the identification of gliadin components encoded by particular *Ae. columnaris* chromosomes [27]. Although extensive polymorphism of electrophoretic spectra of gliadins was demonstrated for durum and common wheat [28], these markers have been broadly exploited for wheat cultivar identification [29] diversity of gliadin profiles of *Aegilops* species, including *Ae. columnaris*, is much lesser studied. Publications were mainly focused on *Ae. tauschii*, the D-genome donor of common wheat [30,31], and only a few papers described other *Aegilops* species [32,33,34].

The aim of the present study was the analysis of intraspecific diversity of *Ae. columnaris* on a broader sample of accessions using cytogenetic (C-banding, FISH with various DNA probes), biochemical (seed storage proteins—gliadins), and molecular (comparative sequence analysis of nuclear and chloroplast DNA fragments) markers.

## 2. Results

### 2.1. C-Banding Analysis of Ae. columnaris

We showed that most of *Ae. columnaris* accessions were karyotypically uniform, but two, K−4224 and a sample provided by Drs. E.A. Nazarova and A.G. Gukasyan in 1998, consisted each of three distinct biotypes, while PI 554187—of two biotypes (Table 1).

**Figure 1:**
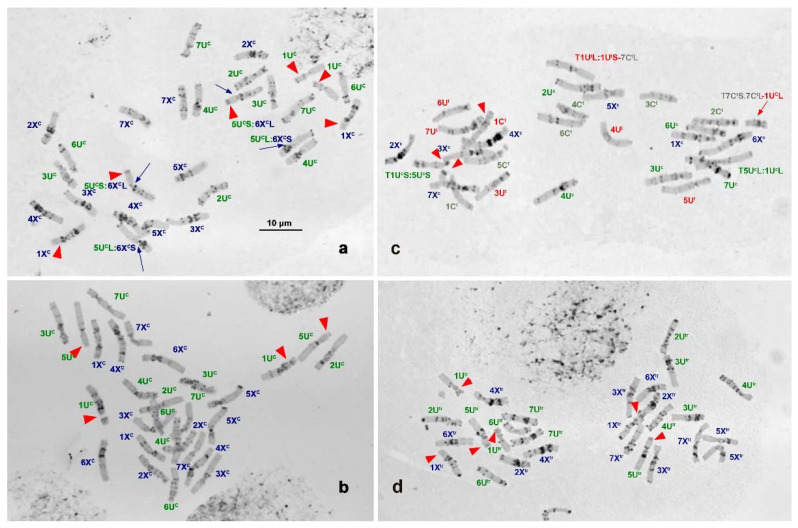
C-banded metaphase cells of accessions representing two karyotypic groups of *Ae. columnaris* (**a**–**c**) in comparison with *Ae. neglecta* (**d**): (**a**)—IG 48818 (C-I); (**b**)—PI 564180 (C-II), (**c**)—the F_1_ hybrid of *Ae. columnaris* (K−4224) × *Ae. triuncialis* (genotype unknown) carrying reciprocal translocations 1U^c^:5U^c^ derived from *Ae. columnaris* and 1U^t^:7C^t^ derived from *Ae. triuncialis*; (**d**)—*Ae. neglecta* (K−4553). Chromosomes are designated according to genetic nomenclature; the U^c^/U^tr^ chromosomes are labeled in dark green, the X^c^/X^tr^ chromosomes in dark blue, the C^t^ of *Ae. triuncialis*—in red, and the U^t^ in grey color). Red arrowheads point to satellite chromosomes. Blue arrows show translocated 5U^c^:6X^c^ chromosomes (**a**).

Three accessions (PI 564182 from Turkey and K−4553 and K−4233 from Armenia) maintained in gene banks under the name *Ae. columnaris* were found to be taxonomically misclassified and were in fact *Ae. neglecta*. Accession IG 49067 was the mix of *Ae. columnaris* and *Ae. biuncialis*, whereas accessions K−2344 from Armenia and AE 1607 of unknown origin—the mix of *Ae. columnaris* and *Ae. triuncialis*. One plant in K−4224 accession was found to be the F_1_ hybrid between *Ae. columnaris* and *Ae. triuncialis* (Figure 1c).

**Figure 2:**
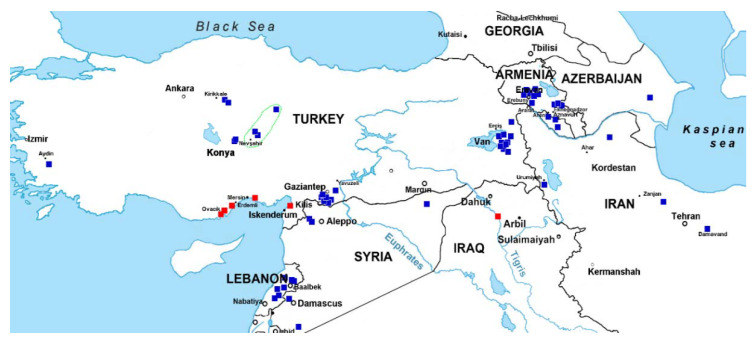
Geographic distribution of the C-I (blue boxes) and C-II (red boxes) accessions of *Ae. columnaris* with known collection sites. Accessions carrying pericentric inversion of 7U^c^ are outlined with a green dotted line.

**Figure 3:**
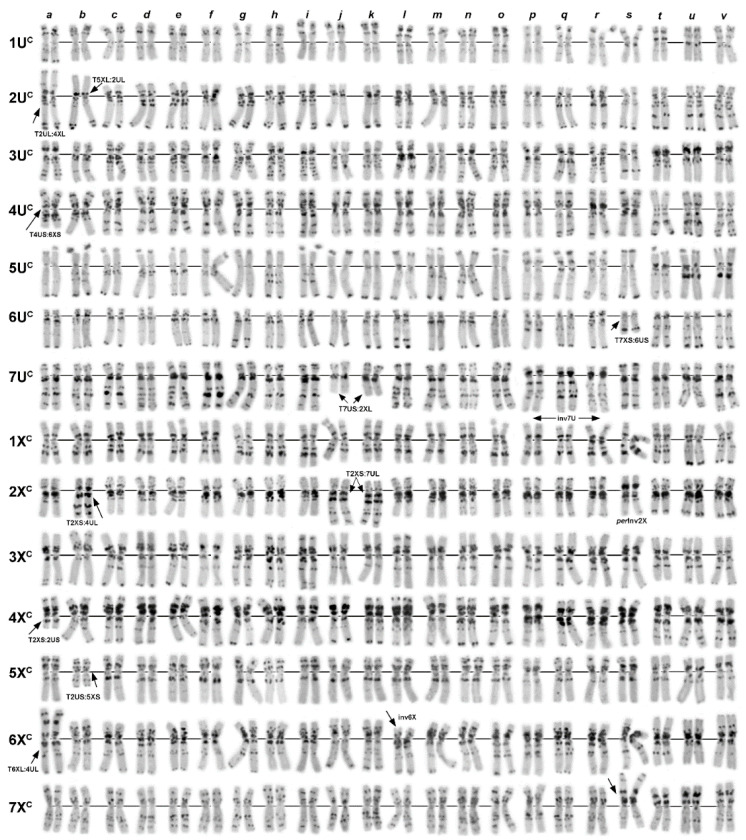
Polymorphisms of the C-banding patterns of Syrian and Lebanese accessions of *Ae. columnaris* (**a**–**s**) in comparison with *Ae. neglecta* (**t**–**v**): (**a**)—K−4372; (**b**)—K−4362; (**c**)—PI 486196; (**d**)—K−2680; (**e**)—PI 487198; (**f**)—K−4009 (Syria); (**g**)—K−4241a; (**h**)—K−4241b; (**i**)—K−4004; (**j**)—K−4003; (**k**)—K−4407; (**l**)—K−4406; (**m**)—K−4007; (**n**)—IG 49067; (**o**)—K−4409 (Lebanon); (**p**)—TX01; (**q**)—Clae34; (**r**)—AE 1521; (**s**)—AE 1607a (unknown origin); (**t**)—PI 564182 (Turkey); (**u**)—K−4233; (**v**)—K−4553 (Armenia). 1U^c^–7X^c^—chromosomes; translocated chromosomes are indicated with arrows and designated, respectively.

**Figure 4:**
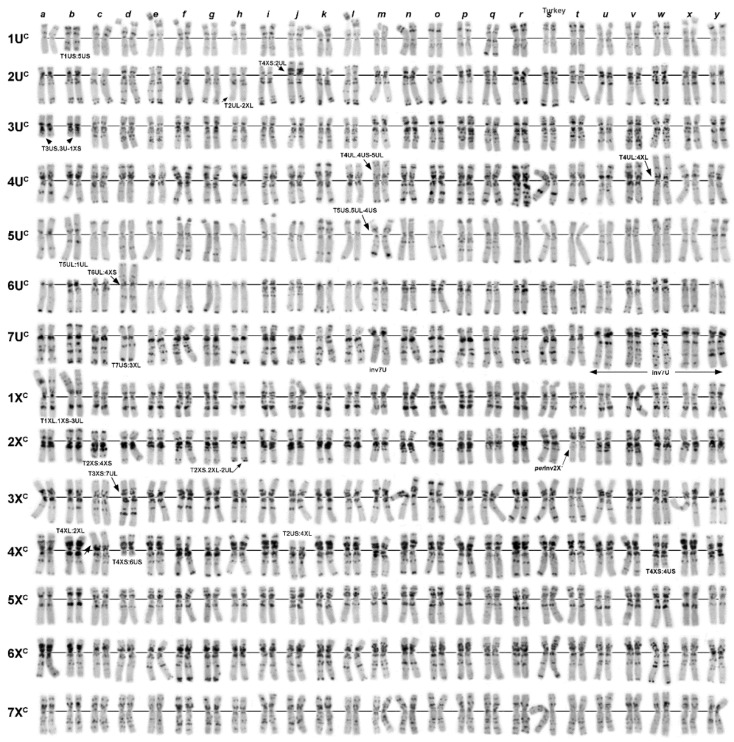
Polymorphisms of the C-banding patterns of Turkish C-I accessions of *Ae. columnaris:* (**a**)—PI 560506; (**b**)—K-PI 554180; (**c**)—PI 560507; (**d**)—PI 486281; (**e**)—PI 554186; (**f**)—PI 554188; (**g**)—PI 554190; (**h**)—PI 554185; (**i**)—PI 554187N; (**j**)—PI 554187T; (**k**)—PI 554178; (**l**)—PI 554186; (**m**)—K−4002; (**n**)—H−6; (**o**)—H−7; (**p**)—H−10; (**q**)—H−3; (**r**)—H−9; (**s**)—H−4; (**t**)—PI 542171; (**u**)—PI 554184; (**v**)—H−8; (**w**)—H−1; (**x**)—i−570045; (**y**)—TA2106. 1U^c^–7X^c^—chromosomes; translocated chromosomes are indicated with arrows and designated, respectively.

**Figure 5:**
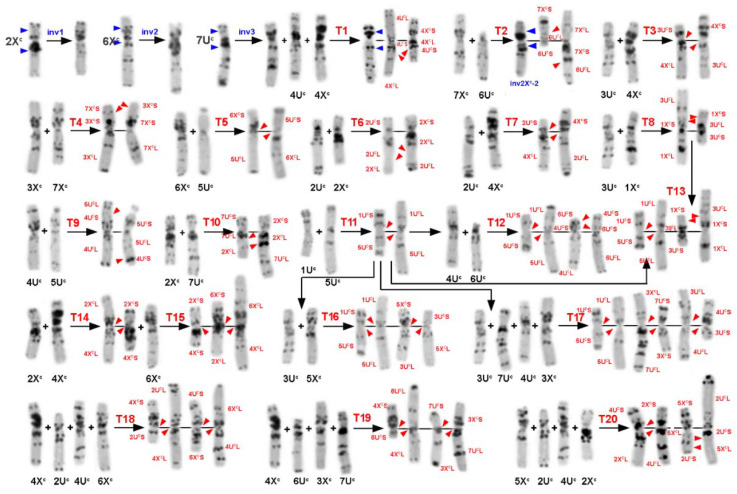
Translocation variants identified among 62 *Ae. columnaris* accessions belonging to group C-I. T1–T20—translocation variants; normal chromosomes are labeled with black letters and are shown below the respective chromosomes; arm combinations in translocated chromosomes are labeled with red letters. Inversions are identified—with blue. Red arrows point to possible translocation breakpoints, and blue arrows point to possible breakpoint positions in inverted chromosomes. Long black arrows define translocation lineages.

**Figure 6:**
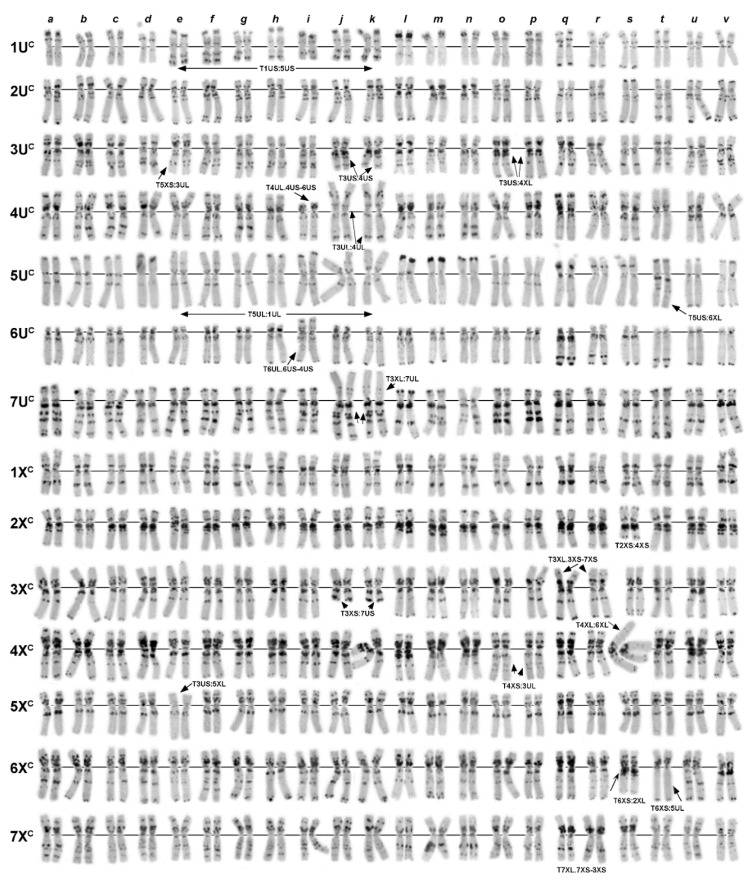
Polymorphisms of the C-banding patterns of Transcaucasian (**a**–**r**) and Iranian (**s**–**v**) accessions of Ae. columnaris: (**a**)—K−2344; (**b**)—K−4224a; (**c**)—K−4229; (**d**)—K−1512; (**e**)—K–4228; (**f**)—K−1178; (**g**)—K−1495; (**h**)—PI 499258; (**i**)—K−1193; (**j**)—K−4225; (**k**)—K−4551; (**l**)—N−1; (**m**)—N−2; (**n**)—N−3; (**o**)—K−4224b; (**p**)—K−564; (**q**)—PI 276457; (**r**)—PI 574457; (**s**)—K−4240; (**t**)—K−4418; (**u**)—IG 48818; (**v**)—K−4413. 1U^c^–7X^c^—chromosomes; translocated chromosomes are indicated with arrows and designated, respectively.

**Figure 7:**
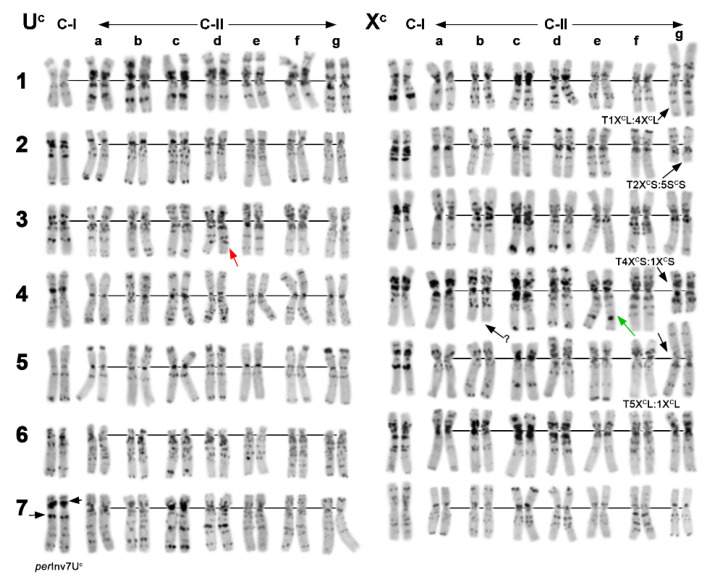
Polymorphisms of the C-banding patterns of *Ae. columnaris* accessions belonging to group C-II in comparison with C-I accession (i−570045). (**a**)—K−3899 (Iraq); (**b**)—PI 542191; (**c**)—PI 564181; (**d**)—PI 554182; (**e**)—PI 564180; (**f**)—PI 564179; (**g**)—TA2084 (all from Turkey). C-I accession i−570045 (=PI 554184) from Turkey is shown for comparison. Rearranged chromosomes are indicated with black arrows. The red arrow indicates a chromosome, which was presumably introgressed from the C-I group; green arrow shows the chromosome, which could be introgressed from *Ae. neglecta*.

The C-banding analysis revealed that *Ae. columnaris* gene pool consists of two distinct karyotypic groups that differed from each other and from *Ae. neglecta* (Figure 1a,b,d) in karyotype structure, the number of satellite chromosomes, and C-banding patterns. The larger group was designated C-I, whereas the smaller one—C-II. Group C-I included 62 accessions collected from an entire distribution range, while C-II comprised only seven accessions, six from the southeast coastal part of Turkey and one from Iraq (Figure 2). Both *Ae. columnaris* groups demonstrated high diversity in the C-banding patterns and, in the case of C-I, broad translocation polymorphism (Figure 3, Figure 4, Figure 5, Figure 6 and Figure 7). Thus, karyotypes of 31 C-I accessions (50%) differed from each other only in the presence/absence or the size of Giemsa C-bands in particular positions; this karyotypic variant was considered basic or “normal (N).” Karyotypes of 31 accessions derived from normal as a result of one or more structural chromosome rearrangements. Accessions collected from geographically closer regions usually had more similar banding patterns than accessions from distant locations, and this trend was also observed in genotypes with chromosomal rearrangements. The highest C-banding and translocation polymorphisms were observed in Turkey.

*Ae. neglecta* was similar to *Ae. columnaris* in the amount and distribution of C-bands on most chromosomes, but differed in the morphology of 6X^c^, which was more metacentric (arm ratio L/S = 1.173 vs. 1.924). In contrast to the C-II group, *Ae. neglecta* carried three pairs of satellite chromosomes as the C-I accessions (Figure 1a,d). Three accessions of *Ae. neglecta* had similar C-banding patterns (Figure 3t–v) and did not possess chromosomal rearrangements.

Twenty-six C-I accessions were collected in different regions of Turkey (Table 1; Figure 4). Nearly half of them (12 accessions) had normal karyotypes (N), and 14 (including segregating accession AE 1607) carried 11 variants of chromosomal rearrangements (Table 2; Figure 4 and Figure 5). Pericentric inversion of the chromosome 7U^c^ (Figure 5, Inv3) was the most frequent variant, which was found in three accessions (Figure 4u,v,x,y). This rearrangement gave rise to a secondary translocation inv7U^c^ + T4U^c^:4X^c^ (T1) identified in the sample H−2 collected in Turkey 132 km NW from Nevşehir (Figure 4w). Double translocation T3X^c^:7U^c^ + T4X^c^:6U^c^—T19 (Figure 4d) was detected in two unrelated accessions, PI 486281 and PI 554181 (Table 2), with identical C-banding patterns. Accession PI 542171 and two AE1607 biotypes carried pericentric inversions of the 2X^c^ chromosome (Figure 4t; Figure 6s), which differed in breakpoint positions resulting in different structures of rearranged chromosomes (Figure 5, Inv1 and inv2X^c^−2). Five translocation variants: T3U^c^:1X^c^ (T8) and its derivative T1U^c^:5U^c^ + T3U^c^:1X^c^ (T13), T2U^c^:2X^c^ (T6), T2U^c^:4X^c^ (T7), T4U^c^:5U^c^ (T9), T3X^c^:7X^c^ (T4), and T2X^c^:4X^c^—T14 (Figure 5; Figure 4a–c,h,j,m, respectively), were found in one accession each (Table 2).

Transcaucasia was represented by 19 Armenian and one Azerbaijani accession (Table 1). Nine accessions had normal karyotypes, and five variants of translocations were identified in the remaining ten accessions (Table 2; Figure 5). Translocation T1U^c^:5U^c^—T10 (Figure 6f,g) was present in five Armenian accessions and in PI 488258 of unknown origin. This translocation gave rise to two double translocations: T1U^c^:5U^c^ + T3U^c^:5X^c^ (T13) T1U^c^:5U^c^ + T4U^c^:6U^c^ (T12) found in one accession each and one triple translocation T1U^c^:5U^c^ + T7U^c^:3X^c^ + T3U^c^:4U^c^—T17 (Figure 6e,i–k) detected in two accessions (Table 2). Interestingly, another complex translocation, the derivative of T1U^c^:5U^c^—T13, was found in Turkey (Figure 4b). The only translocation not related to T1U^c^:5U^c^ was T3U^c^:4X^c^ (T3) identified in two Armenian accessions (Figure 6o,p).

Two of the four Iranian accessions analyzed in a current study carried chromosomal rearrangements (Figure 6s–v). These were a single translocation T5U^c^:6X^c^ (T5) and double cyclic translocation T2X^c^:4X^c^:6X^c^ (T15).

Lebanese group of *Ae. columnaris* contained eight accessions, one of which consisted of two karyotypically normal biotypes differing only in the C-banding patterns (Figure 3g,h). Of them, accession K−4241b (Figure 3h) was almost identical to K−4004 (Figure 3i) in the C-banding pattern. Most Lebanese *Ae. columnaris* had normal karyotypes, and two types of chromosomal rearrangements were identified in three accessions. Thus, K−4003 and K−4407 carried T7U^c^:2X^c^–T10 translocation (Figure 3j,k), while a pericentric inversion of the chromosome 6X^c^ (inv2) was detected in K−4406 (Figure 3l).

Five accessions were from Syria. Three of them had normal karyotypes, and two different complex translocations were identified in the remaining two accessions (Figure 3a,b). K−4372 and K−4362 carried T2U^c^:4X^c^ + T4U^c^:6X^c^ (T18) and T2U^c^:5X^c^ + T4U^c^:2X^c^ (T20), respectively. In both cases, the original single translocations were not found.

The origin of four accessions, AE 1512, AE1607, TX01, and CIae 34, was unknown. We found that AE 1607 consisted of two biotypes differing in chromosomal rearrangements (inv2X^c^/inv2X^c^−2 + T6U^c^:7X^c^) and the C-banding patterns. This accession also contained *Ae. triuncialis* seeds. Three accessions, CIae34, TX01, and AE 1521, carried pericentric inversion of 7U^c^ (Figure 3p–r). This rearrangement was recorded only in *Ae. columnaris* collected from Central Anatolian in Turkey (Figure 2, outlined with green dotted lines); therefore, we suggested that these three accessions may originate from the same region.

Seven *Ae. columnaris* accessions, six from Turkey and one from Iraq, were karyotypically distinct from all other accessions of the species and exhibited significant variation in the C-banding patterns (Figure 7). They were assigned to the C-II group. Accession TA2084 carried at least two whole-arm reciprocal translocations; unidentifiable minor translocations may present in other accessions causing variation in the C-banding patterns. Despite heterogeneity, karyotypes of all C-II accessions shared some distinct features discriminating them from the C-I group and *Ae. neglecta*:

(1) They had only two pairs of the satellite (SAT) chromosomes;

(2) Chromosome 1U^c^ was more heterochromatic;

(3) Chromosome 4U^c^ of C-II contained less heterochromatin compared to C-I (Figure 7);

(4) Chromosome 7U^c^ did not possess a prominent C-band complex in a proximal part of the long arm, which was found in the orthologous chromosomes of all C-I or *Ae. neglecta* accessions.

**Figure 8:**
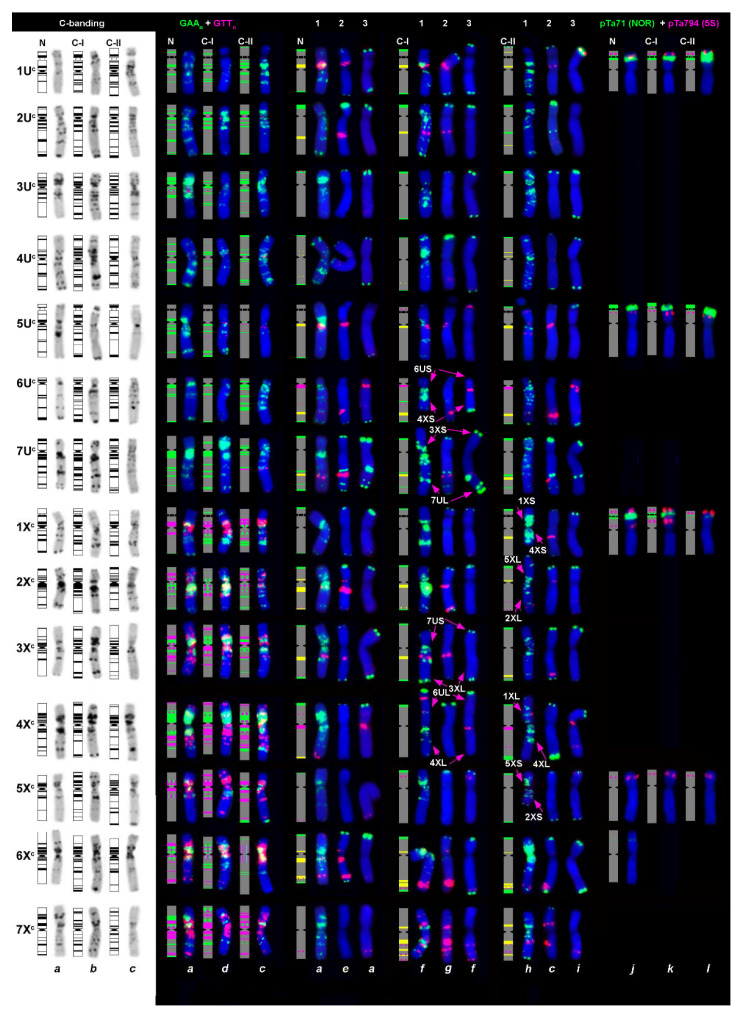
Comparison of the C-banding patterns with the distribution of different types of repeats on chromosomes of Ae. neglecta (N) and C-I and C-II groups of Ae. columnaris. The color of (GAA)_n_, (GTT)_n_, pTa71, and pTa794 probes on the respective idiograms corresponds to their color on chromosomal images. Probe combinations: 1—(GAA)_n_ (green) + pTa−713 (red); 2—pSc119.2 (green) + pTa−713 (red); 3—pSc119.2 (green) + pAs1 (red). Positions of pSc119.2 sites on idiograms are shown in green, pAs1—in pink, and pTa713—in yellow. Accessions codes: (**a**)—PI 564182, (**e**)—K−4233; (**j**)—PI 170209 (Ae. neglecta); (**b**)—H−1 (sample provided by Dr. H. Ozkan); (**d**)—i−570045; (**f**)—PI 554181; (**g**)—K−2680; (**k**)—AE 1607 (Ae. columnaris, C-I); (**c**)—PI 564180; (**h**)—TA2084; (**i**)—PI 564181; (**l**)—PI 542181 (Ae. columnaris, C-II). Arm combinations on rearranged chromosomes are labeled.

Morphology and the C-banding pattern of chromosome 5U^c^ in both C-I and C-II accessions were similar; however, 1U^c^ of C-II was more heterochromatic than the 1U^c^ in C-I (Figure 7 and Figure 8). Significant differences existed in C-banding patterns of other C-I and C-II chromosomes, although some polymorphisms could result from introgression. Thus, chromosome 3U^c^ of PI 554182 (Figure 7d) had the C-banding pattern typical for Turkish and Transcaucasian C-I accessions (i.e., PI 554186, PI 554187 on Figure 4e,i) and may originate via introgression between C-I and C-II groups. A C-banding pattern of chromosome 4X^c^ of PI 564180 was more similar to 4X^t^ of *Ae. neglecta* (Figure 3t–v) than other C-II or C-I accessions.

### 2.2. FISH Analysis of Ae. columnaris

In order to get a deeper insight into genetic differences between groups C-I and C-II of *Ae. columnaris* and to assess their relationship with *Ae. neglecta*, we applied FISH with ribosomal DNA probes pTa71 (45S rDNA), pTa794 (5S rDNA), three microsatellite sequences (GAA)_n_, (GTT)_n_, (ACT)_n_, and three families of the Triticeae-specific satellite DNA sequences pSc119.2, pAs1, and pTa−713. The pTa−535 probe was not considered because it produced signals only on a few chromosomes (Figure S1, *o*; green signals), uninformative for our analyses.

Hybridization of pTa71 and pTa794 probes revealed three pairs of major, nearly equal pTa71 signals on chromosomes of C-I and *Ae. neglecta*, but only two pairs of major NORs in the C-II accessions (Figure 8; Figure S1a,b; Figure S2a,c). Instead, all C-II accessions possessed faint pTa71 signals on a chromosome pair carrying a clear distal 5S rDNA locus. This chromosome was classified as 1X^c^ based on results of sequential FISH with 5S + 45S rDNAs followed by (GAA)_n_ + (GTT)_n_/pTa−713 probes (Figure S2b,d). An additional minor NORs were found in the middle of 6U*L (Figure S3) of all *Ae. columnaris* and *Ae. neglecta* accessions. *Ae. neglecta* differed from *Ae. columnaris* in the presence of a minor 45S rDNA site in a distal part of an arm of a pair of large metacentric X*-genome chromosome tentatively designated as 6X^t^ (Figure 8; Figure S1c, arrowed; Figure 2e; Figure S3a). The application of FAM-labeled oligo-probes allowed us to detect very weak minor pTa71-signals at the terminus of 5X*L, a distal quarter of 1U*L, and in a proximal part of 3X^c^S (Figure S3). Similar signals were obtained on chromosomes of the C-II accession PI 564181 (data not shown). However, these minor sites never appeared when the plasmid DNA was used as a probe, and they were not considered in the analysis.

Apparent differences between C-I, C-II groups, and *Ae. neglecta* existed in the pattern of 5S rDNA probe. All *Ae. columnaris* C-I and *Ae. neglecta* accessions contained ten 5S rDNA signals distributed among four chromosome pairs (Figure S2a,e; Figure S3). The chromosome 1X^*^ possessed two pTa794 sites: one located distally to the NOR, while the second—proximally to it (Figure 8; Figure S1a,c; Figure S2a,e). By contrast, four chromosome pairs in all C-II accessions carried a single 5S rDNA signal each.

In *Ae. columnaris* and *Ae. neglecta* labeling patterns of (GAA)_n_ probe were largely consistent with the C-banding patterns, while the (GTT)_n_ hybridized predominantly on the X^c^ chromosomes (Figure S1g–i). Only 2U*, 4U*, and 5U* contained small (GTT)_n_ sites in pericentromeric/ proximal regions, and a faint signal was present in the middle of the 7U^c^L arm (Figure 8) in four of the five C-II accessions. By contrast, all X^c^ genome chromosomes demonstrated prominent (GTT)_n_ signals located predominantly in the proximal chromosome regions. Positions of the (GTT)_n_ clusters on the X^c^ chromosomes only partially overlapped with the (GAA)_n_ locations; some chromosomes (e.g., 5X^c^ or 7X^c^) that were poorly labeled with (GAA)_n_, showed extremely heavy labeling with (GTT)_n_ (Figure 8). Hybridization patterns of (ACT)_n_ probe were almost identical to that of (GTT)_n_ (Figure S1m,n).

The pSc119.2 probe hybridized to subtelomeric regions of one or both arms of most *Ae. columnaris* chromosomes except for 7X^c^, which lacks pSc119.2 signals in all C-I and most C-II accessions (Figure 8; Figure S1j,k,n,o). Intercalary sites appeared only on the long arm of 7U^c^ and rarely on 6U^c^L, as in a diploid *Ae. umbellulata*. Labeling patterns of the pSc119.2 probe were polymorphic between and among C-I and C-II accessions (Figure 8). Four of the five C-II accessions studied by FISH possessed intercalary pSc119.2 site also on the chromosome 2U^c^L (Figure 8c), but this site was never observed in C-I or *Ae. neglecta*. On the other side, the C-II accession PI 564181 did not possess any pSc119.2 signals on chromosome 2U^c^ (Figure 8i).

The D-genome specific probes pAs1 and especially pTa−535 were not very informative for chromosome identification in *Ae. columnaris* and *Ae. neglecta*. Distinct pAs1 sites were observed in the pericentromeric region of 6U^*^ and 4X* chromosomes of all studied species, whereas 2–3 weak signals were present on 4X*S and 7X^*^L arms. The chromosome 5X^c^ of C-I also contained a single, small pAs1 site in the distal half of the short arm. Hybridization sites of the pTa−535 probe emerged on the 6U^c^L arm, but only in a few accessions studied (Figure S1o; Figure S3b).

The pTa−713 probe hybridized to most *Ae. columnaris* (C-I and C-II) and *Ae. neglecta* chromosomes, while 3U^c^, 4X^c^, and 5X^c^ (in *Ae. neglecta*—also 7X^t^) lacked the signals completely. In most cases, the distribution of pTa−713 sites on chromosomes of all three groups was similar; however, some differences between them were observed (Figure 8; Figure S4). In particular, a large pTa−713 signal was present on the short arm of 1U^c^ of all C-I and *Ae. neglecta* accessions, but it was absent in the C-II. Most C-I and one *Ae. neglecta* (K−4233) possessed a distinct site in the proximal half of 2U^c^L, which was not found in C-II and two *Ae. neglecta* accessions (Figure S4g,h). We did not observe proximal pTa−713 sites in the short arm of 2X^c^L and 3X^c^L in the C-II group, but they were present in all C-I and *Ae. neglecta* accessions. A large pTa−713 signal present on the 1X^c^L arm of all C-II accessions was never observed in the C-I group or *Ae. neglecta* (Figure S4). Position of hybridization sites on 5U^c^, 6U^c^, and 7U^c^, was similar in all three groups, but *Ae. columnaris* differed from *Ae. neglecta* in morphology and/ or labeling patterns of chromosomes 6X^*^ and 7X* (Figure 8).

### 2.3. Analysis of Gliadin Spectra of Ae. columnaris

**Figure 9:**
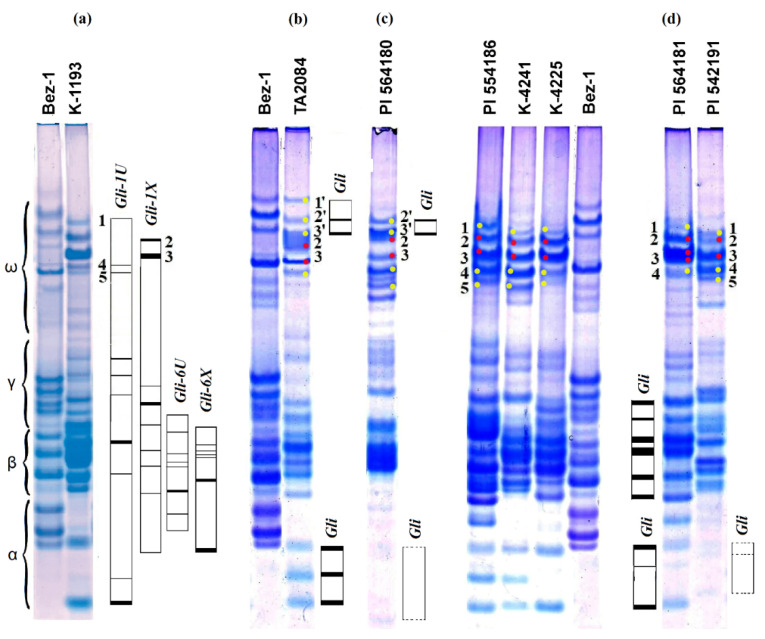
*Ae. columnaris* with the most distinct gliadin spectra**:** (**a**) gliadin spectrum of a model *Ae. columnaris* accession K−1193 in comparison with etalon spectrum of wheat cultivar Bezostaya−1 (Bez−1). Blocks of linked electrophoretic gliadin components controlled by a single locus of the particular *Ae. columnaris* chromosome [27] are shown schematically at the right side of the electrophoretic spectrum; (**b**) EP spectrum of the accession TA2084 in comparison with wheat cultivar Bezostaya−1; (**c**) EP spectrum of PI 564180; (**d**) EP spectra of *Ae. columnaris* accessions illustrating protein components presumably encoded by the X^c^ (red dots) and U^c^ (yellow dots) chromosomes. The unique components, which were not found in any other *Ae. columnaris* accessions, are shown schematically (parts (**b**–**d**)).

Electrophoretic analysis revealed a high diversity of gliadin spectra in 25 *Ae. columnaris* accessions and their distinctness from the spectra of *Ae. neglecta* (Figure S5). Only two of 25 *Ae. columnaris* accessions, K−4413 and K−4418 from Iran, shared similar gliadin spectra, whereas four C-II accessions included in our analysis were highly diverse. However, all contained electrophoretic (EP) components, whose position did not match the overall pattern specific for *Ae. columnaris* C-I accessions (Figure 9).

Thus, electrophoretic profiles of PI 564180 and PI 542191 were characterized by low-intense, virtually invisible (“minor”) components in the α-zone; their intensities and position were distinct from other accessions of *Ae. columnaris* (Figure 9c,d; Figure S5). Based on comparison with the K−1193 spectrum, we proposed that these components can be encoded by both the X^c^ and U^c^ genomes (Figure 9).

Protein components located in the ω-zone of the spectra of all *Ae. columnaris* C-I accessions were similar in intensity and position (Figure S5). Among them, components designated as “2” and “3” (Figure 9, indicated with red dots) corresponded to components detected in the spectrum of K−1193, which were coded by chromosome 1X^c^. In contrast to other materials, accession PI 564181 contained the unique double band instead of “component 3” (Figure 9d). In addition, it displayed a distinct distribution of components located in the β–γ zone, which, by comparison with the K−1193 spectrum, can be coded by group-6 chromosomes of the U^c^ and X^c^ genomes. Such distribution was more typical for common wheat, and the respective zone was controlled by wheat chromosomes 6B and 6D [35].

Protein components encoded by chromosome 1U^c^ were characterized by low intensities (Figure 9a,c; indicated by yellow dots). By contrast, the spectra of TA 2084 and PI 564180 possessed several intense components in the upper part of ω-zone designated 1′, 2′, and 3′. By comparing with the spectrum of K−1193, we hypothesized that they could be controlled by the chromosome 1U^c^ (Figure 9b,c). TA 2084 and PI 564180 spectra shared components 2′ and 3′with similar mobility and intensity, but they differed in the presence of additional minor component 1′, which showed slower mobility in TA2084.

### 2.4. Variability of the U-Genome Specific U31 Nuclear Fragment in Ae. columnaris and Ae. neglecta

**Figure 10:**
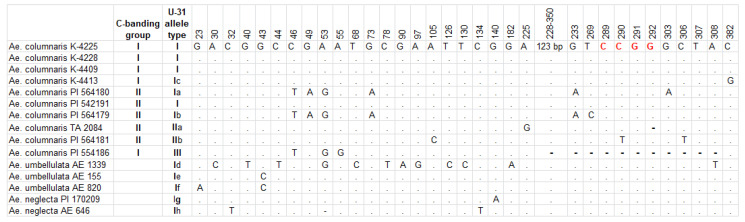
Nucleotide substitutions in the U31 region in 15 *Ae. columnaris* (U^c^U^c^X^c^X^c^), *Ae. neglecta* (U^t^U^t^X^t^X^t^), and *Ae. umbellulata* (UU) sequences. Dots correspond to nucleotides identical to consensus sequences. The *Msp*I restriction site is highlighted in red.

Amplification and further sequencing of the U-genome-specific U31 nuclear fragment was performed with primers U31a and U31b in 15 accessions, including ten *Ae. columnaris* (K−4225, K−4228, K−4409, K−4413, and PI554186 from different countries and representing chromosomal group C-I, and PI542191, PI564179, PI 564180, PI564181, and TA2084 all from Turkey representing group C-II), two *Ae. neglecta* from Algeria and Turkey (PI 170209 and AE 646) in comparison with three accessions of their diploid parental species *Ae. umbellulata (*AE 1339, AE 155, and AE 820) of different geographic origin (Table 1). All accessions analyzed generated 363 bp fragments, except for *Ae. columnaris* PI 554186. In this accession, the fragment length was reduced to 270 bp due to a 123 bp deletion (Figure 10; Figure S6).

The sequence of the U31 fragment obtained from *Ae. columnaris* accessions fall into three types, which corresponded to designations proposed earlier by Kadosumi et al. [23] based on fragment length and the presence of *Msp*I restriction site (CCGG). Type-I having the full-length U31 fragment and an intact *Msp*I site was found in seven *Ae. columnaris* accessions as well as in all analyzed *Ae. neglecta* and *Ae. umbellulata* accessions (Figure S6; Figure 10).

The type-II U31 fragment was identified in two *Ae. columnaris* accessions, both from the C-II chromosomal group (Figure 10). It emerged as a result of sequence changes at the *Msp*I restriction site: a mononucleotide deletion in position 292 was found in TA2084, while C/T_290_ substitution in PI 564181. Accession PI 554186 (C-I) possessed the type-III U31 fragment with a 123 bp deletion (Figure S6). All U31-alleles assigned to type-II corresponded to those reported by Kadosumi et al. [23] in *Ae. columnaris* or *Ae. neglecta*. Among U31 type-I accessions of *Ae. columnaris*, four allelic variants were found, three of which were novel alleles (Figure 10). Two of them were identified in C-II and one in C-I accession.

The U31 sequences of *Ae. umbellulata* accessions AE 155 and AE 820 and both *Ae. neglecta* accessions (PI 170209 and AE 646) belonged to type-I and showed just a few (1–2) nucleotide substitutions, while almost 12 SNPs were detected in the U31 sequence of *Ae. umbellulata*, AE 1339 from Greece, which was also assigned to type-I (Figure S6). Most of the U31 alleles of *Ae. umbellulata* or *Ae. neglecta* discovered in this study (Figure 10) were not identified earlier, and only *Ae. neglecta* accession PI 170209 carried the same allele as *Ae. columnaris* (KU−2953A) from Armenia, described earlier by Kadosumi et al. [23].

**Figure 11:**
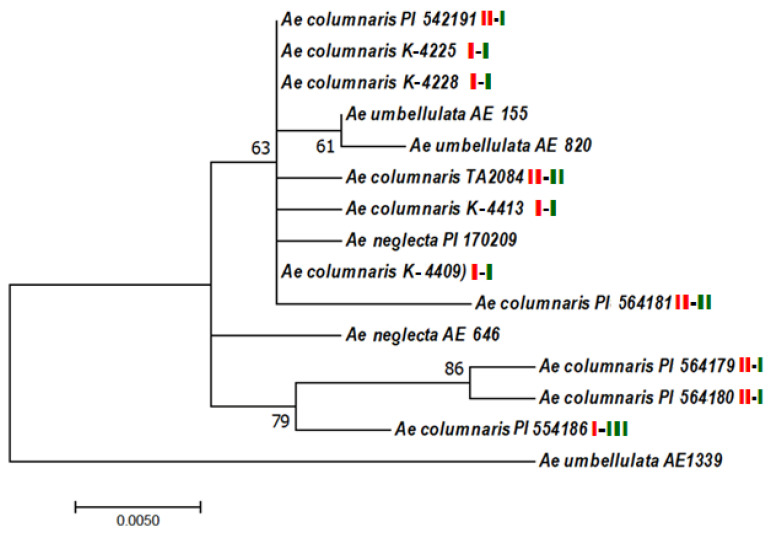
Maximum-likelihood (Kimura 2-parameter model) tree of the U-genome-specific U31 nuclear sequence. The numbers above the branches indicate bootstrap values; the C-banding group is shown in red, U31 allele type—in green.

An ML tree (Figure 11) shows the possible evolutionary relationship between accessions and species based on comparative sequencing of the U31 alleles. All *Aegilops* accessions except AE 1339 (*Ae. umbellulata*) formed one common cluster on the tree obtained. No species-specific or ploidy-specific clusters have been observed. Three *Ae. columnaris* accessions including two of type-II U31 alleles (PI 564179 and PI 564180) and one type-III accession (PI 554186) formed a separate sub-cluster with 79% bootstrap support. Other accessions representing different species (*Ae. columnaris* and *Ae. neglecta*) and different U31 allele types (I and II) fall into one common sub-cluster with *Ae. umbellulata* (AE 115 and AE 820) showing a closer relationship. *Ae. neglecta* accession (AE 646) form an individual branch.

### 2.5. Variability of Three Plastome Intergenic Spacers in Ae. columnaris and Ae. neglecta

**Figure 12:**
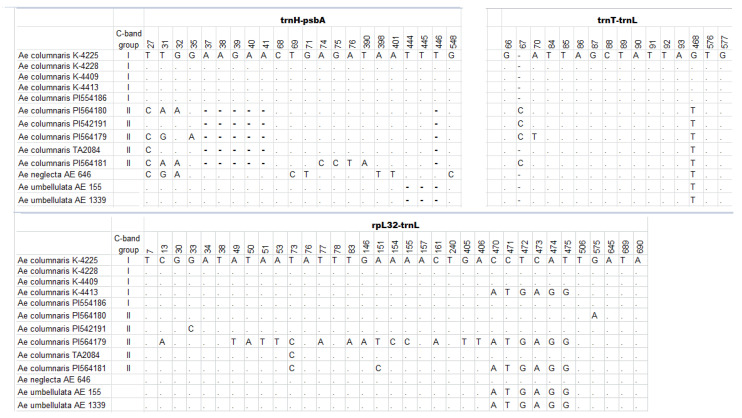
Nucleotide substitutions in *trnH-psbA*, *trnT-trnL*, *rpL32-trnL* plastome regions in 13 *Ae. columnaris* (U^c^U^c^X^c^X^c^), *Ae. neglecta* (U^t^U^t^X^t^X^t^), and *Ae. umbellulata* (UU) sequences. Dots correspond to nucleotides identical to consensus sequences.

Variability of three plastome fragments, *trnH(gtg)*-*psbA*, *trnT(ugu)-trnL(uaa)*, and *rpL32-trnL(tag)* DNA, were assessed on the same set of 10 *Ae. columnaris* accessions as for nuclear U31 fragment. The total length of plastome sequences obtained corresponded to 1825 bp (*trnH-psbA*—558 bp, *trnT-trnL*—577 bp, and *rpL32-trnL*—690 bp). Polymorphism levels differed between the analyzed fragments: only three SNPs were found in the *trnT-trnL* spacer, while *rpL32-trnL* and *trnH-psbA* sequences were much more polymorphic. In contrast to *Ae. columnaris*, spacer sequences of two *Ae. umbellulata* accessions (AE 155 and AE 1339) were invariable (Figure 12).

According to the analysis of all three plastome regions, 10 *Ae. columnaris* accessions split into two groups (plastogroups). Four C-I accessions (K−4225, K−4228, K−4409, and PI 554186) had identical sequences of the plastome spacers, while K−4413 (C-I, Iran) differed at a single site: substitution of the hexamer sequence CCTCAT by ATGAGG at position 470−475 of the *rpl32-trnL* spacer (Figure 12). Accessions in group C-II, PI542191, PI564179, PI 564180, PI564181, and TA2084, showed significantly higher sequence polymorphisms at all three plastome regions. Nevertheless, they all shared the same deletion of one of the two AAGAA 5-bp repeats, as well as the deletion of the mononucleotide T at position 446. (Figure 12). In addition, they all carried G/T substitution at position 468 of *trnT-trnL*, as *Ae. neglecta* and *Ae. umbellulata* accessions.

C-II accession PI 564179 possessed the highest number of mutations, especially in the *rpL32-trnL* sequence. Together with C-II accession PI 564181 and Iranian C-I K−4413, it carried ATGAGG/CCTCAT sequence substitution. The same substitution was also identified in *Ae. umbellulata* and *Ae. neglecta* (Figure 12). Comparison of the observed plastogroups with groups discriminated based on C-banding and FISH analyses showed that all *Ae. columnaris* accessions characterized by an increased variability (PI542191, PI564179, PI 564180, PI564181, and TA2084) belonged to group C-II, while low polymorphic accessions (K−4225, K−4228, K−4409, K−4413, PI 554186) fall to C-I.

On the ML tree (Figure S7), all *Ae. columnaris* accessions with invariable plastome sequences clustered together, whereas K−4413 formed a separate branch in a common sub-cluster with two *Ae. umbellulata* accessions (bootstrap = 67). Five genetically variable *Ae. columnaris* accessions fall either in a common sub-group with *Ae. neglecta* (TA2084, PI 542191, and PI 564180), or formed separate branches (PI 564179, PI 564181) (Figure S7).

## 3. Discussion

Cytogenetic (C-banding, FISH), biochemical (seed storage proteins—gliadins), and molecular (sequence analysis of polymorphic U31 nuclear fragment and three intergenic regions of cpDNA) analyses showed close genetic relationships between *Ae. columnaris* and *Ae. neglecta*, which is in agreements with previous studies [2,4,11,12,15,19,23]. From the other hand, chromosome analysis revealed higher genetic diversity of *Ae. columnaris* compared to that reported for *Ae. neglecta* [4,13,23], which was expressed in higher C-banding/FISH-polymorphisms and broader spectra of chromosomal rearrangements as well as by a higher number of U31 alleles and higher variability of cpDNA identified in these species.

Two karyotypic groups, C-I and C-II, have been discriminated within *Ae. columnaris* based on chromosome analysis and each group displayed characteristic C-banding and FISH patterns. Group C-I was mainly similar to *Ae. neglecta*, whereas C-II differed from the C-I group of *Ae. columnaris* and *Ae. neglecta* in karyotype structure, heterochromatin distribution, and in the patterns of rDNA loci. Such heterogeneity of ribosomal loci was not reported for other *Aegilops* species [13,36,37,38,39]. Although these karyotypic groups were not supported by comparing gliadin profiles or sequences of the U31 nuclear fragment, they fully agreed with plastogroups discriminated based on cpDNA analysis.

Groups C-I and C-II karyotypically differed from each other, but the divergence level varied between individual chromosomes. Thus, no significant changes were observed in 2U^c^, 5U^c^, 2X^c^, and 6X^c^, while 3U^c^, 4U^c^, 7U^c^, 1X^c^, 5X^c^, and 7X^c^ of the C-II were modified. Despite it, we found chromosomes among C-II accessions which matched chromosomes of C-I (e.g., 3U^c^ of PI 554182) or *Ae. neglecta* (e.g., 4X^c^ of PI 564180), which can be caused by introgressions. Another evidence of gene flow between species and chromosomal groups came from the analysis of the U31 nuclear fragment: Type-II U31 allele identified in C-II accession PI 564181 (Figure 12) was earlier detected by Kadosumi et al. [23] in four accessions of *Ae. neglecta* and three *Ae. umbellulata*, but not in *Ae. columnaris*. A similar trend was observed in the presence of ATGAGG/CCTCAT substitution in the *rpl32-trnL* spacer region, which was present in one C-I and two C-II accessions of *Ae. columnaris*, but also in *Ae. neglecta* and *Ae. umbellulata* (Figure 12).

All methods used in our study highlighted significant genetic diversity in both C-I and C-II chromosomal groups, but each of them exhibited a different type of polymorphism. Karyotype divergence in the C-I group was associated with variation in the presence and size of C-bands in particular positions and chromosomal rearrangements identified here in 55% of the accessions studied. However, no polymorphisms that could be associated with introgressions or unbalanced rearrangements have been found. The results of electrophoretic analysis of seed storage proteins led to the same conclusion. Although 25 accessions of *Ae. columnaris* had unique gliadin profiles, the spectra of most C-I genotypes shared several characteristic bands, especially in the α-zone. The number of U31 alleles identified here in the C-I accessions (Figure 10) was relatively small, and this group displayed very low polymorphism in the intergenic spacers of cpDNA; only one 6-bp-substitution in position 470 of *rpl32-trnL* was found (Figure 12).

By contrast, accessions constituting the C-II group were highly heterogeneous. Although karyotypes of all accessions carried several diagnostic features discriminating them from the C-I group and *Ae. neglecta*, the observed variation cannot be explained by polymorphism of heterochromatic regions only. Some variants can be due to introgressions and heterochromatin re-patterning. In contrast to group C-I, chromosomal rearrangements did not play such an essential role in the divergence of the C-II group: translocations were detected only in TA2084, which is geographically distant from others (Table 1; Figure 2). However, minor translocations may exist in other C-II accessions, but they cannot be identified due to the lack of appropriate markers. Significant heterogeneity of the C-II group was also shown by gliadin analysis. All four C-II accessions had different gliadin profiles, which did not possess any common components. The spectra of each of the C-II accessions (PI 564180, PI 564181, TA2084, and PI 542191), however, carried a number of features (band loss or gain; bands that differed in intensity or position) which were not observed in the C-I group.

The comparative sequence analysis of the U31 nuclear fragment and three plastome intergenic spacer regions also revealed the highly heterogeneous composition of the C-II groups. Thus, the U31 fragment of type-I was found in three C-II accessions (Figure 10), but two of them carried mutant alleles. All accessions with type-II U31 fragment belonged to the C-II group. It was an interesting observation because, according to Kadosumi et al. [23], type-II U31 fragment occurred extremely rare in *Ae. columnaris*, although frequently in *Ae. umbellulata*. Both type-II alleles identified here in the C-II accessions corresponded to those described earlier by these authors, but they found one allele in *Ae. columnaris* from Syria, while the second—in *Ae. neglecta*. Kadosumi et al. [23] also identified an additional type-II U31 allele, not found in this work, in *Ae. columnaris* from Iran; however, the karyotypic group of this accession was not determined.

In contrast to the relatively conservative C-I group, from three to 27 SNPs covering all three intergenic spacer regions of cpDNA were identified among accessions of the C-II group.

An interesting fact uncovered by molecular analysis of the U31 nuclear fragment was an unexpectedly high number of SNPs (12) identified in *Ae. umbellulata* accession AE 1339 from Greece (Figure S6), which showed no changes in the cpDNA (Figure 12). According to FISH [40], this accession was karyotypically normal and similar to other *Ae. umbellulata* genotypes in the distribution of repetitive DNA probes [4,41,42,43,44,45,46]. All these indicated that the observed mutations in AE 1339 were not caused by chromosomal rearrangement. From the other hand, Kawahara [47] has already uncovered the distinctness of *Ae. umbellulata* population from Greek Islands based on morphological and isozyme markers.

Summarizing our results, we can conclude that *Ae. columnaris* is phylogenetically very close to *Ae. neglecta*, and probably derived from this species (or their common ancestor). It is supported by the following observations.

Owing to a species-specific inversion in chromosome 6X^c^, the karyotype of *Ae. columnaris* becomes more “asymmetric” compared to *Ae. neglecta*. According to Stebbins [48], an increase in karyotype asymmetry is a trend of evolution in plant species and, therefore, *Ae. neglecta* karyotype should be considered “more primitive”, while *Ae. columnaris*—“more advanced”;

1. The chromosome 6X^t^ of *Ae. neglecta* possesses a minor 45S rDNA locus, which probably pre-existed in the progenitor *Aegilops* species; however, this locus is absent in *Ae. columnaris*.
2. *Ae. columnaris* is characterized by chromosome instability expressed in a higher proportion and broader diversity of chromosomal rearrangements (20 variants in more than 55% of accessions). Chromosome instability is an essential factor of speciation [49,50] and is usually more expressed in phylogenetically new species. In addition, we found significant intraspecific polymorphism in *Ae. columnaris* plastome, although the only low variation of the chloroplast DNA sequences was recorded in *Triticum* and *Aegilops* species [51,52].

The similarity of rDNA and repetitive DNA patterns of chromosomes of *Ae. neglecta* and group C-I of *Ae. columnaris* and their distinctness from chromosomes of the C-II accessions indicate that the C-I group diverged from *Ae. neglecta* or their common ancestor as a result of minor genome modifications. Group C-II could derive from a progenitor presumably belonging to group C-I of *Ae. columnaris* relatively recently, probably due to introgression from another *Aegilops* species, accompanied by significant reorganization of the parental genomes. As most C-II accessions with known collection sites originated from a very narrow geographic region of the southeastern coastal part of Turkey (Figure 2, red boxes), they might originate from one common ancestor. Significant heterogeneity of the C-II accessions in karyotype structure, C-banding, and FISH patterns, gliadin composition, and nuclear and chloroplast DNA sequences may indicate that they are currently at the initial stage of species divergence; most likely, this group is undergoing an extensive speciation process.

## 4. Materials and Methods

Intraspecific diversity of *Aegilops columnaris* Zhuk. (2*n* = 4*x* = 28, U^c^U^c^X^c^X^c^) was assessed on a set of 69 accessions of various geographic origin in comparison with the related tetraploid species *Ae. neglecta* Req. ex Bertol. (2*n* = 4*x* = 28, U^t^U^t^X^t^X^t^) —four accessions and *Ae. umbellulata* Zhuk. (2*n* = 2*x* = 14, UU), the diploid U-genome progenitor of *Ae. columnaris* and *Ae. neglecta*—three accessions. All 69 accessions were analyzed using C-banding, while 16 *Ae. columnaris*, three *Ae. neglecta*, and two *Ae. umbellulata* accessions were studied by FISH. Gliadin profiles were examined on 25 *Ae. columnaris* accessions of various geographic origins and one *Ae. neglecta* (Table 1), whereas 10 *Ae. columnaris* (five from C-I and five from C-II groups), two *Ae. neglecta* and three *Ae. umbellulata* accessions were selected for subsequent molecular analysis.

The materials were obtained from the gene banks of the N.I. Vavilov Institute of Plant Genetic Resources, S.-Petersburg, Russia; USDA-ARS (Aberdeen, Idaho, USA); Wheat Genetics and Genomics Resource Centre (WGGRC), Kansas State University, Kansas, USA; and Leibniz Institute of Plant Genetics and Crop Plant Research (IPK), Gatersleben, Germany. One sample of *Ae. columnaris* was collected by Drs. E.A. Nazarova and A.G. Gukasyan (Erevan, Armenia) during an expedition of Takhtadjan Institute of Botany (1997) near the village Urznadzor, Armenia. Nine samples were collected in 2005–2006 by Dr. H. Özkan during expeditions to different regions of Turkey.

### 4.1. DNA Probes

The following DNA probes were used:

1. pTa71 was used as plasmid DNA (a 9 kb long sequence of common wheat encoding 18S, 5.8S and 26S rRNA genes including spacers [53] or the 5′ FAM-end-labeled (Syntol, Moscow, Russia) oligo-probe 5′-GGG CAA AAC CAC GTA CGT GGC ACA CGC CGC CTA-3′ [54];
2. pTa794 was used as plasmid DNA (a 420 bp long sequence of wheat containing the 5S rRNA gene and intergenic spacer [55] or as the 5′ Cy−3-end-labeled (Evrogen, Moscow, Russia) oligo-probe 5′-TCA GAA CTC CGA AGT TAA GCG TGC TTG GGC GAG AGT AGT AC-3′ [56];
3. pSc119.2—a 120 bp long sequence isolated from rye [57];
4. pAs1—a 1 kb fragment derived from *Ae. tauschii* and belonging to *Afa* family [58];
5. pTa535−1 was used as 5’ 6-carboxyfluorescein (6-FAM) or 6-carboxytetra-methylrhodamine (TAMRA) end-labeled (MWG, Germany) oligo-probe (5′-AAA AAC TTG ACG CAC GTC ACG TAC AAA TTG GAC AAA CTC TTT CGG AGT ATC AGG GTT TC-3′) [54,59];
6. pTa−713 was used as 5′6-carboxytetra-methylrhodamine (TAMRA) or Cy3 end-labeled oligo-probe (5′-GTC GCG GTA GCG ACG ACG GAC GCC GAG ACG AGC ACG TGA CAC CAT TCC CAC CCT GTC TA-3′) [54,59];
7. The oligo-(GTT)_9_ probe labeled at the 3′-end with fluorescein−12-dUTP was synthesized in the laboratory of biological microchips at the Engelhardt Institute of Molecular Biology, Moscow, Russia.
8. The oligo-(GAA)_10_ probe labeled at the 3′-end with fluorescein−12-dUTP or Cy3 was synthesized in the laboratory of biological microchips at the Engelhardt Institute of Molecular Biology, Moscow, Russia.
9. The oligo-(ACT)_10_ probe labeled at the 3′-end with Cy3 was synthesized in the laboratory of biological microchips at the Engelhardt Institute of Molecular Biology, Moscow, Russia.

### 4.2. Giemsa C-Banding Method

The C-banding procedure was carried out as described in Badaeva et al. [60]. Chromosomes of *Ae. columnaris* were classified according to genetic nomenclature developed earlier by Badaeva et al. [20] based on analysis of introgressive lines. Chromosomes of *Ae. neglecta* were classified according to similarity with *Ae. columnaris* chromosomes. Designation of *Ae. umbellulata* chromosomes followed the nomenclature suggested by Friebe et al. [41].

### 4.3. Fluorescence In Situ Hybridization

FISH was carried out according to the protocol described in Badaeva et al. [61]. The probes labeled with fluorescein were detected using anti-fluorescein/Oregon green^®^, rabbit IgG fraction, Alexa Fluor^®^ 488 conjugate (Molecular Probes, Eugene, OR, USA). The slides were counter-stained with DAPI (4′,6-diamidino-2-phenylindole) in Vectashield mounting media (Vector Laboratories, Peterborough, UK) and examined on a Zeiss Imager D−1 microscope. Selected metaphase cells were captured with AxioCam MRm digital camera using software AxioVision, version 4.6. Images were processed in Adobe Photoshop^R^, version CS5 (Adobe Systems, Edinburgh, UK).

### 4.4. Seed Storage Protein (Gliadin) Analysis

Electrophoresis (EP) in polyacrylamide gel (PAG) according to the previously published protocol [62] was employed to obtain gliadin spectra of the 25 *Ae. columnaris* and one *Ae. neglecta* accessions. The spectra of wheat cultivar Bezostaya−1 (a standard for gliadin spectra of common wheat) and *Ae. columnaris* K−1193 with the known genetic control of gliadin components [27] were used to compare gliadin profiles of other *Aegilops* accessions (Figure 9a).

### 4.5. DNA Extraction, PCR Amplification, and DNA Sequencing

Ten accessions of *Ae. columnaris* (five C-I representing five countries and five C-II from Turkey), *Ae. umbellulata* (3 accessions) and *Ae. neglecta* (2 accessions) were selected for analyses by molecular methods. Genomic DNA was extracted from 10-day-old seedlings using the DNeasy Plant Mini kit (QIAGEN, Hilden, Germany). DNA quantitative and qualitative evaluation was performed using NanoDrop 2000c spectrophotometer (ThermoFisher-Scientific, Madison, WI, USA).

Amplification of the U-genome-specific U31 nuclear fragment was performed using primers U31a and U31b [23] with PCR conditions: an initial denaturation step of 95 °C for 5 min followed by 30 cycles of 94 °C for 1 min, 55 °C for 1 min, and 72 °C for 1 min with a final extension step at 72 °C for 3 min. The amplified fragments were sequenced directly from both ends with the same U31a and U31b primers.

Amplification of the three intergenic spacers regions (*trnH(ugu)-psbA, rpl32-trnL(tag), trnT(ugu)-trnL(uaa)*) of the plastome DNA of *Aegilops* accessions was performed using primer sets listed in Table S1. PCR amplification was performed in a 15 μL reaction mixture containing approximately 50 ng genomic DNA, 1.5 μL of 10× PCR buffer, 1.5 mM MgCl_2_, 0.2 mM of dNTPs, 0.3 μM of each primer, and 0.5 unit of *Taq* DNA polymerase. The PCR conditions were as follows: an initial denaturation step of 95 °C for 5 min, followed by 30 cycles of 94 °C for 1 min, annealing at the appropriate Tm for 1 min, and 72 °C for 1 min with a final extension step at 72 °C for 5 min. Annealing temperatures for *trnH-psbA* was 58 °C; *trnL-rpl32*—56 °C; and *trnT-trnL*—55 °C. The same primers were used to sequence the obtained chloroplast DNA fragments; PCR products were cleaned before sequencing using the QIAquick PCR purification kit (QIAGEN, Hilden, Germany). PCR products were sequenced using standard protocols with the ABI Prism Big Dye Terminator cycle sequencing kit v. 3.1. Sequences were resolved on an ABI Prism 3100 automated sequencer.

A phylogenetic tree was constructed based on U31 data and combined chloroplast sequence data using MEGA 7 [63] based on ML (maximum likelihood) method. Kimura 2-parameter model was used for U31 and Tamura−3 parameter model for cpDNA, which was selected using Modeltest; 1000 bootstrap replicates were applied for the branch support evaluation. The SNP data from 10 *Ae. columnaris* genotypes were taken for subsequent analyses. The SNP position was determined from the first nucleotide of U31 or of each of the analyzed chloroplast spacers.

## Figures and Tables

**Table 1:** The list of materials studied and their origins.

No	Accession #	Duplicates	Country of Origin	Collection Site	Latitude (N)	Longitude (E)	Alt. (h, m)	Analyzed by FISH
1 ^1^	K−1178	-	Armenia	Aznaburt vil., near Dash Agl mountain	39.4333	45.2833	1600	-
2 ^1^	K−1193	-	Armenia	Abovyan, near village of Shor-Bulakh	40.1333	45.6333	1300	+
3 ^1^	K−1495	IG 48026	Armenia	Naxchivan, Djulfinskii reg., N of Arbakunis vil.	39.1167	45.6333	1350	-
4	K−1512	AE 1188	Armenia	near Erevan, valley of Razdan river	40.2	44.5333	136	-
5 ^1^	K−2344	-	Armenia	Ekhegnadzor reg., along Elpin-Agavnadzor road	39.7833	45.1833	776	-
6 ^1^	K−4224 (3)	IG 48738	Armenia	Erevan region, 2 km SE of Jrvezh	40.1667	44.6	780	-
7 ^1,2^	k−4228	IG 48757	Armenia	Shorap, 20 km W of Erevan	40.25	44.3333	1350	-
8	K−4229	IG 126249	Armenia	Abovyan reg., Erebuni Natural Reserve	40.2833	44.6333	1072	-
9 ^1,2^	K−4225	IG 48740	Armenia	Outskirt NE Erevan	40.1167	44.5167	1400.0	-
10 ^1^	K−4366	IG 48745	Armenia	Abovyan reg., NE of Erevan, N. Dzervesh; Gegadir; Muchavan	40.2833	44.6333	1045	-
11 ^1^	K−4551	-	Armenia	Vanadzor, after Gadzor	39.7833	45.3667	363	-
12	K−564	-	Armenia	Azizbekovskii reg., around vil. Khandzorut	39.55	45.35	1685	+
13	NAZ (3)	-	Armenia	near the village of Urznadzor	-	-	-	-
14	PI 499258	-	unknown	obtained from China	-	-	-	-
15	PI 574457	K−512; AE111	Azerbaijan	unknown	-	-	-	-
16	IG 48818	-	Iran	Damavand	35.7333	52.0667	2474	-
17 ^1^	K−4240	IG 49138	Iran	10 km SW Horand from Ahar	38.75	47.1667	1110	-
18 ^1,2^	K−4413 *	IG 49087	Iran	20 km W Takestan to Zia Abad road to Zanjan	36.0333	49.5	1320	-
19	K−4418	IG 49107	Iran	31 km Urumiyeh to Oshnaviyeh Kazem Lo Valley	37.25	45.1333	1380	-
20	K−3899 ^II^	IG 49010	Iraq	Ninawa; Jebel Maqloub near Deir Matti	36.5	43.4167	850	-
21	IG 49067	-	Lebanon	Rachaiya, 1 km E of Aita Al Foukhar	33.6333	35.9	1350	-
22	K−4003	IG 48072	Lebanon	Terbol region W slope Anti Lebanon Mts. (zone A)	33.9	36.1	-	-
23	K−4004	IG 48091	Lebanon	Sanin region E slope Lebanon Mts. (zone B)	33.9333	35.8333	-	-
24	K−4007	IG 48107	Lebanon	Irsal region; W slope Anti Lebanon Mts. (zone C)	34.25	36.6667	0	-
25 ^1^	K−4406	IG 49047	Lebanon	Baalbek 4 km W Baalbek road to Bcharre laat vil.	34.0333	36.1667	1050	-
26 ^1^	K−4241a	i−611188	Lebanon	Sanin region E slope Lebanon Mts. (zone B)	33.9333	35.8333	-	-
27 ^1^	K−4241b	i−611189	Lebanon	Al Alia; 40 km N of Karak	31.95	35.9333	800	-
28	K−4407	IG 49047	Lebanon	Baalbek 4 km W Baalbek road to Bcharre Iaat vil.	34.0333	36.1667	1050	-
29 ^2^	K−4409	IG 49053	Lebanon	3 km from Deir Ahmar road to Ain Ata	34.1333	36.1	1370	-
30 ^1^	K−2680	PI 487198	Syria	7 km from Atareb to Qalaat Samaan, Aleppo	36.2022	36.7758	460	-
31	K−4009	i−571713	Syria	Al Hasakah; just N of Jabal Abd El-Aziz	36.4667	40.3333	600	+
32 ^1^	K−4362	IG 48729	Syria	Damascus May Saloun; 4 km before Tukeya	33.6	36.0667	1468	-
33 ^1^	K−4372	IG 48800	Syria	2 km NE of Sa’an road from Shabki	32.7	36.8417	1400	-
34	PI 487196	-	Syria	Aleppo Province	36.1667	36.8333	450	-
35	CIae 34	-	Turkey	-	-	-	-	-
36	K−4002	IG 47875	Turkey	14 km NW Keskin	39.7167	33.4333	520	-
37	#1	2006−6−25−8−2	Turkey	132 km NW from Nevşehir	39.1622	33.9325	1060	-
38	#10	2006−6−21−5−1	Turkey	50 km NE from Kilis to Gaziantep	37.315	37.7347	510	-
39	#2	2006−6−17−7−2	Turkey	29 km NE from Kilis to Gaziantep	36.9242	37.0786	730	-
40	#3	2006−6−21−12−1	Turkey	57 km NE from Kilis to Gaziantep	37.2678	37.5208	700	-
41	#4	2006−7−12−2	Turkey	34 km NE from Kilis to Gaziantep	37.09667	37.0406	970	-
42	#6	2006−6−21−9−1	Turkey	54 km NE from Kilis to Gaziantep	37.3739	37.8458	700	-
43	#7	2006−6−17−10−2	Turkey	32 km NE from Kilis to Gaziantep	36.9944	37.9664	950	-
44	#8	2006−6−25−6−3	Turkey	114 km NW from Nevşehir	39.0442	34.0414	880	-
45	#9	2006−6−21−1−2	Turkey	46 km NE from Kilis to Gaziantep	37.2139	37.4903	831	-
46 ^1^	i−570045	PI 554184	Turkey	Kars, 5 km S Sivas Malatya border	39.7333	37.05	1500	+
47	PI 276968	-	Turkey	Konya	37.8333	32.5	-	-
48	PI 486281	IG 46886	Turkey	42 km southeast of Ercis-Karayollari Bakimevi	38.9167	43.6	1700	+
49^1^	PI 542171	-	Turkey	19 km north of Gaziantep toward Yavuzeli	37.1833	37.4667	800	+
50 ^1,2^	PI 542191 ^II^	-	Turkey	Aegean Agric. Research Inst. Gene Bank, Menemen	-	-	30	+
51	PI 554178	IG 47040	Turkey	22 km north of Van	38.7	43.3333	1734	+
52	PI 554180	IG 46997	Turkey	35 km west of Tuzluca	40.15	43.3667	1010	-
53	PI 554181	IG 47042	Turkey	Aydin, 10 km north of Kusadasi	37.9167	27.2833	130	+
54	PI 554182 ^II^	IG 47048	Turkey	Icel, 24 km southwest of Erdemli, Mersin across from Boy Scout Recreation Center	36.4667	34.1333	30	-
55	PI 554185	IG 47117	Turkey	2 km southeast of Van on route to Gurpinar	38.5	43.3667	1790	-
56 ^1,2^	PI 554186	IG 47166	Turkey	Van, 6 km southeast of Van	38.4667	43.3833	1990	-
57 ^1^	PI 554187 (2)	IG 47125	Turkey	Van, 2 km north of Van	38.5333	43.3333	1710	-
58	PI 554188	-	Turkey	Van, 29 km north of Van	38.75	43.3667	1790	-
59	PI 554190	IG 47170	Turkey	Van, 29 km north of Van	38.75	43.3667	-	-
60	PI 560506	-	Turkey	Roadside along Lake Van. About 3 km W of Ermisler	38.8667	43.4667	1630	-
61	PI 560507	-	Turkey	Van, About 2 km N of village of Yalnizagac	38.7	43.5	1837	-
62 ^2^	PI 564179 ^II^	-	Turkey	23 km southeast of Manavgat, near Okucalar village	36.6833	31.6333	50	+
63^1,2^	PI 564180 ^II^	-	Turkey	58 km southwest of Silifke; 4 km west of Ovacik, Mersin	36.18333	33.6333	250	+
64 ^1,2^	PI 564181 ^II^	-	Turkey	49 km southwest of Silifke, Mersin	36.2	33.7	150	+
65 ^1,2^	TA 2084 ^II^	-	Turkey	1 km N of Iskenderum (Alexandretta)	36.6001	36.1969	50	+
66	TA 2106	KU11−2	Turkey	Konya, collected by Dr. Johnson in 1965.	37.8667	32.4833	1030	-
67	AE 1521	-	unknown	-	-	-	-	-
68	AE 1607 (2)	-	unknown	obtained from UK	-	-	-	+
69	TX 01	-	unknown,	provided by Dr. M. Feldman	-	-	-	-
	*Aegilops neglecta*							
70	PI 564182 *	-	Turkey	9 km southeast of Ayvacik	39.583333	26.483333	420	+
71 ^1^	K−4553 *	IG 126975	Armenia	Kapan distr. road from Kapan to Charaten	39.1903	46.43	970	-
72 ^2^	PI 170209	-	Turkey	17 km south of Canakkale	40.033333	26.35	100	+
73 ^2^	AE 646	-	Algeria	unknown	-	-	-	+
	*Aegilops umbellulata*							
74 ^2^	AE 155	K−1234	Azerbaijan	unknown	-	-	-	+
75 ^2^	AE 820	-	Turkey	3 km E Kemalpasa	-	-	-	-
76 ^2^	AE 1339	-	Greece	Kreta	-	-	-	+

Column 1 (No): ^1^—accessions used for electrophoretic analysis; ^2^—accessions used for molecular analysis. Column 2 (Accession #): *—accessions that were erroneously classified as *Ae. columnaris*; ^II^—accessions belonging to group II.

**Table 2:** Variants of chromosomal rearrangements identified in *Ae. columnaris*, type I accessions.

No.	Trans. Code	Translocation Type	Structure of Translocated Chromosomes	Accessions	Origin
1	inv1	*per*Inv2X^c^	-	PI 542171; AE 1607b	Turkey
2	inv2	*per*Inv 6X^c^	-	K−4406	Lebanon
3	inv3	*per*Inv 7U^c^	-	i−570045; CIae 34; K−4002; TA2106; AE 1521; H−8; TX01	Turkey
4	T1	*per*Inv7U^c^ + T4U^c^:4X^c^	4U^c^S:4X^c^S + 4U^c^L:4X^c^L	H−2	Turkey
5	T2	*per*Inv2X^c^−2 + T6U^c^:7X^c^	*per*Inv2X^c^−2 + 6U^c^S.6U^c^L−7X^c^S +T6U^c^L−7X^c^S.7X^c^L	AE 1607a	unknown
6	T3	T3U^c^:4X^c^	3U^c^S:4X^c^L + 3U^c^L:4X^c^S	K−4224c; K−564	Armenia
7	T4	T3X^c^:7X^c^	3X^c^L.3X^c^S−7X^c^S + 3X^c^S−7X^c^S.7X^c^L	PI 276968 PI 574457 (K−512)	Turkey Azerbaijan
8	T5	T5U^c^:6X^c^	5U^c^S:6X^c^L + 5U^c^S:6X^c^L	K−4418	Iran
9	T6	T2U^c^:2X^c^	2U^c^S.2U^c^L−2X^c^L + 2U^c^L−2X^c^L.2X^c^S	PI 554185	Turkey
10	T7	T2U^c^:4X^c^	2U^c^S:4X^c^L + 2U^c^S:4X^c^L	PI 554187t	Turkey
11	T8	T3U^c^:1X^c^	3U^c^S.3U^c^L−1X^c^ + 3U^c^L−1X^c^S.1X^c^L	K−560506	Turkey
12	T9	T4U^c^:5U^c^	4U^c^L.4U^c^S−5U^c^L + 4U^c^S−5U^c^L.5U^c^S	K−4002	Turkey
13	T10	T7U^c^:2X^c^	7U^c^S. 7U^c^L−2X^c^ + 7U^c^L−2X^c^L.2X^c^S	K−4003; K−4407	Lebanon
14	T11	T1U^c^:5U^c^	1U^c^S:5U^c^S + 1U^c^L:5U^c^L	PI 499258; K−1178; K−1495; K−4224; K−4366	Armenia
15	T12	T1U^c^:5U^c^ + T4U^c^:6U^c^	1U^c^S:5U^c^S + 1U^c^L:5U^c^L + 4U^c^L.4U^c^S−6U^c^S + 4U^c^S−6U^c^S.6U^c^L	K−1193	Armenia
16	T13	T1U^c^:5U^c^ + T3U^c^:1X^c^	1U^c^S:5U^c^S + 1U^c^L:5U^c^L + 3U^c^S.3U^c^L−1X^c^ + 3U^c^L−1X^c^S.1X^c^L	PI 554180	Turkey
17	T14	T2X^c^:4X^c^	2X^c^S:4X^c^S + 2X^c^L:4X^c^L	PI 560507	Turkey
18	T15	T2X^c^:4X^c^:6X^c^	2X^c^S:4X^c^S + 2X^c^L:6X^c^S + 6X^c^L:4X^c^L	K−4240	Iran
19	T16	T1U^c^:5U^c^ + T3U^c^:5X^c^	1U^c^S:5U^c^S + 1U^c^L:5U^c^L + 3U^c^S:5X^c^L + 3U^c^L:5X^c^S	K−4224B; K−4228	Armenia
20	T17	T1U^c^:5U^c^ + T7U^c^:3X^c^ + T3U^c^:4U^c^	1U^c^S:5U^c^S + 1U^c^L:5U^c^L + 7U^c^S:3X^c^S + 7U^c^L:3X^c^L + 3U^c^S:4U^c^S + 3U^c^L:4U^c^L	K−4225; K−4551	Armenia
21	T18	T2U^c^:4X^c^ + T4U^c^:6X^c^	2U^c^S:4X^c^S + 2U^c^L:4X^c^L + 4U^c^S:6X^c^S + 4U^c^L:6X^c^L	K−4372	Syria
22	T19	T6U^c^:4X^c^ + T7U^c^:3X^c^	6U^c^S:4X^c^S + 6U^c^L:4X^c^L + 7U^c^S:3X^c^L + 7U^c^L:3X^c^S	PI 486281; PI 554181	Turkey
23	T20	T2U^c^:5X^c^ + T4U^c^:2X^c^	2U^c^L.2U^c^S−5X^c^ + 2U^c^S−5X^c^L.5X^c^S + 4U^c^S:2X^c^L + 4U^c^L:2X^c^S	K−4362	Syria

## Data Availability

The data presented in this study are available in Supplementary Material, Figure S6.

## Supplementary Materials

The following are available online at https://www.mdpi.com/article/10.3390/plants10050956/s1, Figure S1. Hybridization of different DNA probes on chromosomes of C-I (a,d,g,j,n,o) and C-II (b,e,h,k,m) groups of *Ae. columnaris* in comparison with *Ae. neglecta*—T (c,f,i,l,m). Chromosomal group, accession numbers, and probe combinations are given on corresponding cell images; the labeling of probe color corresponds to signal color. Chromosomes are numbered according to genetic nomenclature; the U^c^ chromosomes are labeled with yellow, while the X^c^–white letters. Scale bar–10 µm. Figure S2. Sequential FISH with 5S (red) and 45S (green) rDNA probes (a,c,e) followed by hybridization with (GAA)n (green) and pTa-713 (red) (b,d,f) on chromosomes of *Ae. columnaris* accessions AE 1607 (C-I, (a,b)), PI 542191 (C-II, (c,d)), and *Ae. neglecta* accession PI 564182. Chromosomes are designated according to genetic nomenclature, the Uc chromosomes are labeled with white and Xc with yellow letters. Translocated T6Uc:7Xc chromosomes are indicated with red arrowheads (b); white arrows point to the positions of minor NORs on chromosome 1Xc (c). Green arrows point to *mi*nor NORs on chromosome 6Xtr (f). Owing to a limited space of metaphase images, the superscripts “c” and ‘tr’ are omitted in the figure. Figure S3. Location of minor NORs on chromosomes of *Ae. neglecta*, PI 564182, by sequential FISH with oligo-probes pTa71-1 (green) and pTa794 (red)–(a), followed by (GAA)n (red) and pAs1 (green)–(b). Minor NORs are indicated with arrows (a), and the respective chromosomes are identified according to their (GAA)n-FISH patterns (b). Figure S4. Diversity of the pTa-713 probe patterns on chromosomes of *Ae. columnaris*, C-I (a–f) in comparison with *Ae. neglecta*, T (g,h) and *Ae. columnaris*, C-II (i–l): (a)–AE 1607; (b)–K-4241; (c)–K-1193; (d)–i-570045); (e)–PI 486281; (f)–PI 554178; (g)–PI 564182; (h)–IG 170209; (i)–PI 542191; (j)–PI 564181; (k)–TA2084; (l)–PI 564180. Chromosomes are classified based on pSc119.2 (a,l) or (GAA)n-labelling patterns (b–k), green signals. Hybridization sites of pTa-713 probe are visualized in red. Chromosomes are classified according to genetic nomenclature; translocated chromosomes are designated. Invariable pTa-713 sites are underlined with yellow dotted lines. Figure S5. Diversity of gliadin spectra of *Ae. columnaris* accessions collected from different countries in comparison with *Ae. neglecta* (N). Accession numbers are shown on the top, while their geographic origin—on the bottom of the figure. α, β, γ, and ω—zones in electrophoretic spectra. Figure S6. Nucleotide variability in the U31 region of analyzed Aegilops accessions. The *MspI* restriction site is highlighted in yellow. Figure S7. Maximum-Likelihood (Tamura-3 parameter model) phylogenetic tree of *combined trnH-psbA, trnT-trnL, rpL32-trnL* sequences; the numbers above the branches indicate bootstrap values: branch length was measured in a number of substitutions per site. Table S1. Primers for amplification and sequencing of plastome fragments.
