# Supplementary material for: Chromosome and Molecular Analyses Reveal Significant Karyotype Diversity and Provide New Evidence on the Origin of Aegilops columnaris
Source: Plants (Basel). 2021 May 11;10(5):956. doi: 10.3390/plants10050956 (PMC8151338; doi:10.3390/plants10050956)
Supplement: Supplementary file 1 [file plants-10-00956-s001.zip › plants-1201575-supplementary.pdf]

Original Research article

Chromosome and molecular analyses reveal significant karyotype diversity and provide new evidence on the origin of *Aegilops columnaris*

Ekaterina D. Badaeva[1,2*], Nadezhda N. Chikida[3], Andrey N. Fisenko[1], Sergei A. Surzhikov[2], Maria Kh. Belousova[3], Hakan Özkan[4], Alexandra Yu. Dragovich[1], and Elena Z. Kochieva[5]

[1] – N.I.Vavilov Institute of General Genetics, Russian Academy of Sciences. Gubkina street 3, GSP-1, Moscow 119991, Russia

[2] – Engelhardt Institute of Molecular Biology, Russian Academy of Sciences. Vavilova street 34, GSP-1, Moscow 119991, Russia

[3] - Federal Research Center, N.I. Vavilov All-Russian Institute of Plant Genetic Resources, Bolshaya Morskaya street 44, St. Petersburg 190121, Russia

[4] – Department of Field Crops, Faculty of Agriculture, University of Çukurova, 01330 Adana, Turkey

[5] - Federal Research Center “Fundamentals of Biotechnology” of the Russian Academy of Sciences. 60 let Oktjabrya prospect 7, build. 1, Moscow 117312, Russia

* - corresponding author: Badaeva Ekaterina D., e-mail: katerinabadaeva@gmail.com; tel: +7 499 135-0460; Fax: +7 499 132 8962

Supplementary materials:

Supplementary Table 1. Primers for amplification and sequencing of plastome fragments

Plastome fragment	Primers 5’-3’	Annealing temperature
(*trnH(ugu)-psbA*	F: CGC GCA TGG TGG ATT CAC AAT CC<br>R: GTT ATG CAT GAA CGT AAT GCT C	58 ^{0}C
rpl32-trnL(tag)	F: CAGTTCCAAAAAAACGTACTTC<br>R: CTGCTTCCTAAGAGCAGCGT	56 ^{0}C
trnT(ugu)-trnL(uaa)	F: TCT ACC GAT TTC GCC ATA TC<br>R: CAA ATG CGA TGC TCT AAC CT	55 ^{0}C

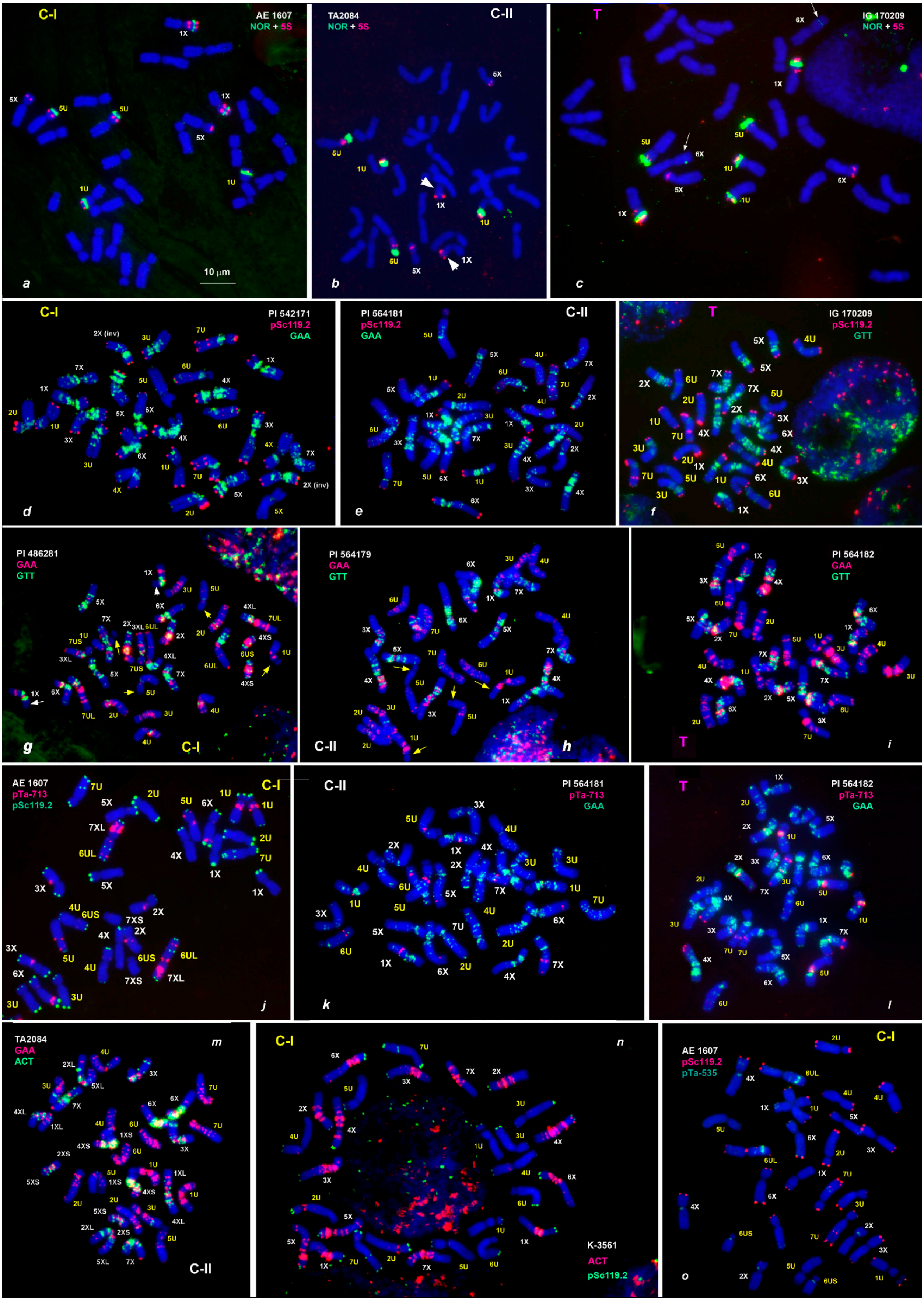

Supplementary Figure S1. Hybridization of different DNA probes on chromosomes of C-I (*a, d, g, j, n, o*) and C-II (*b, e, h, k, m*) groups of *Ae. columnaris* in comparison with *Ae. neglecta* – T (*c, f, i, l, m*). Chromosomal group, accession numbers, and probe

combinations are given on corresponding cell images; the labeling of probe color corresponds to signal color. Chromosomes are numbered according to genetic nomenclature; the U^c chromosomes are labeled with yellow, while the X^c – white letters. Scale bar – 10 μm.

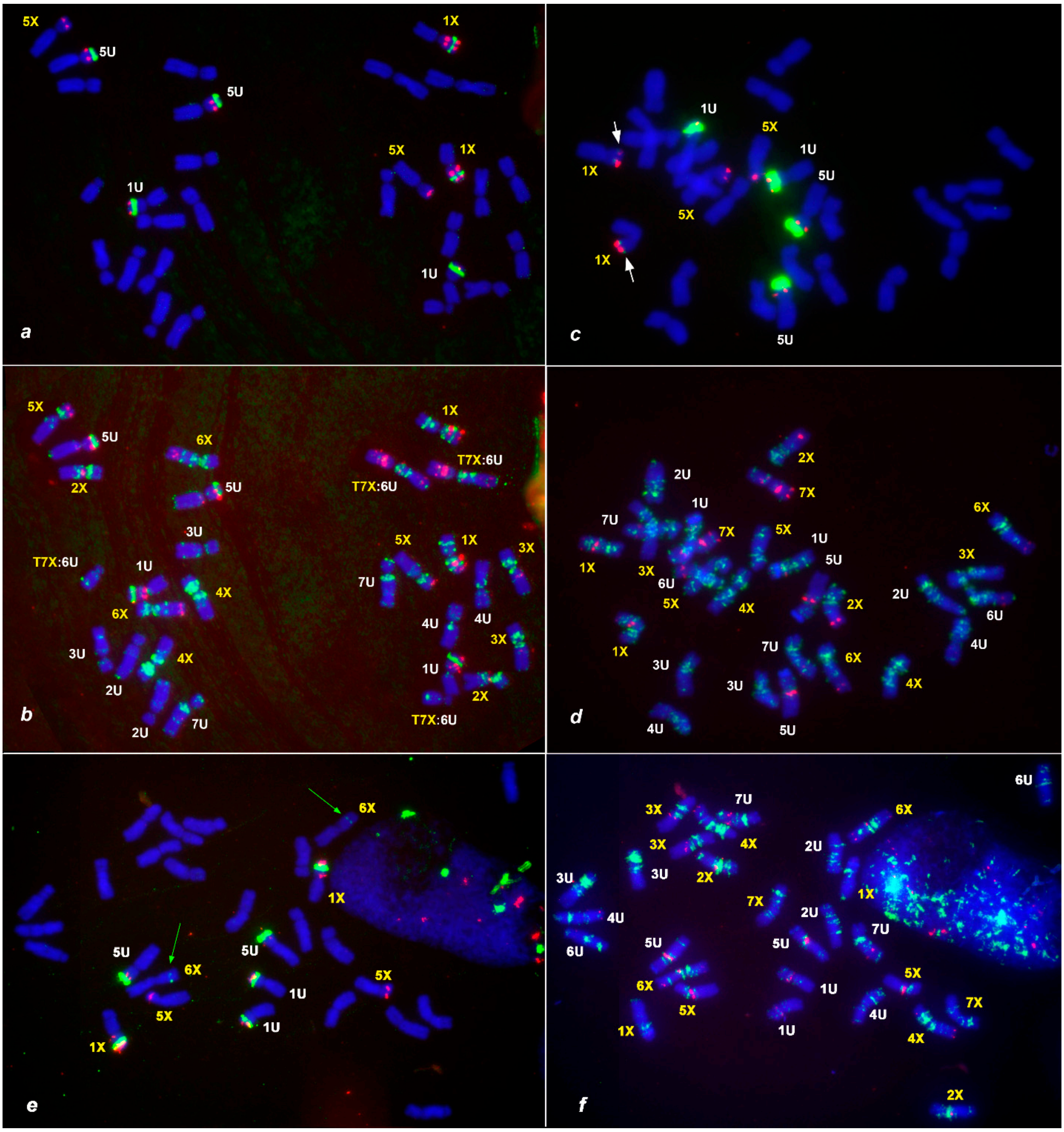

Supplementary Figure S2. Sequential FISH with 5S (red) and 45S (green) rDNA probes (*a, c, e*) followed by hybridization with $(GAA)_n$ (green) and pTa-713 (red) (*b, d, f*) on chromosomes of *Ae. columnaris* accessions AE 1607 (C-I, *a, b*), PI 542191 (C-II, *c, d*), and *Ae. neglecta* accession PI 564182. Chromosomes are designated according to genetic nomenclature, the U^c chromosomes are labeled with white and X^c with yellow letters. Translocated $T6U^c$:$7X^c$ chromosomes are indicated with red arrowheads (*b*); white arrows point to the positions of minor NORs on chromosome $1X^c$

(*c*). Green arrows point to minor NORs on chromosome $6X^{tr}$ (*f*). Owing to a limited space of metaphase images, the superscripts “^{c}” and ‘^{tr}’ are omitted in the figure.

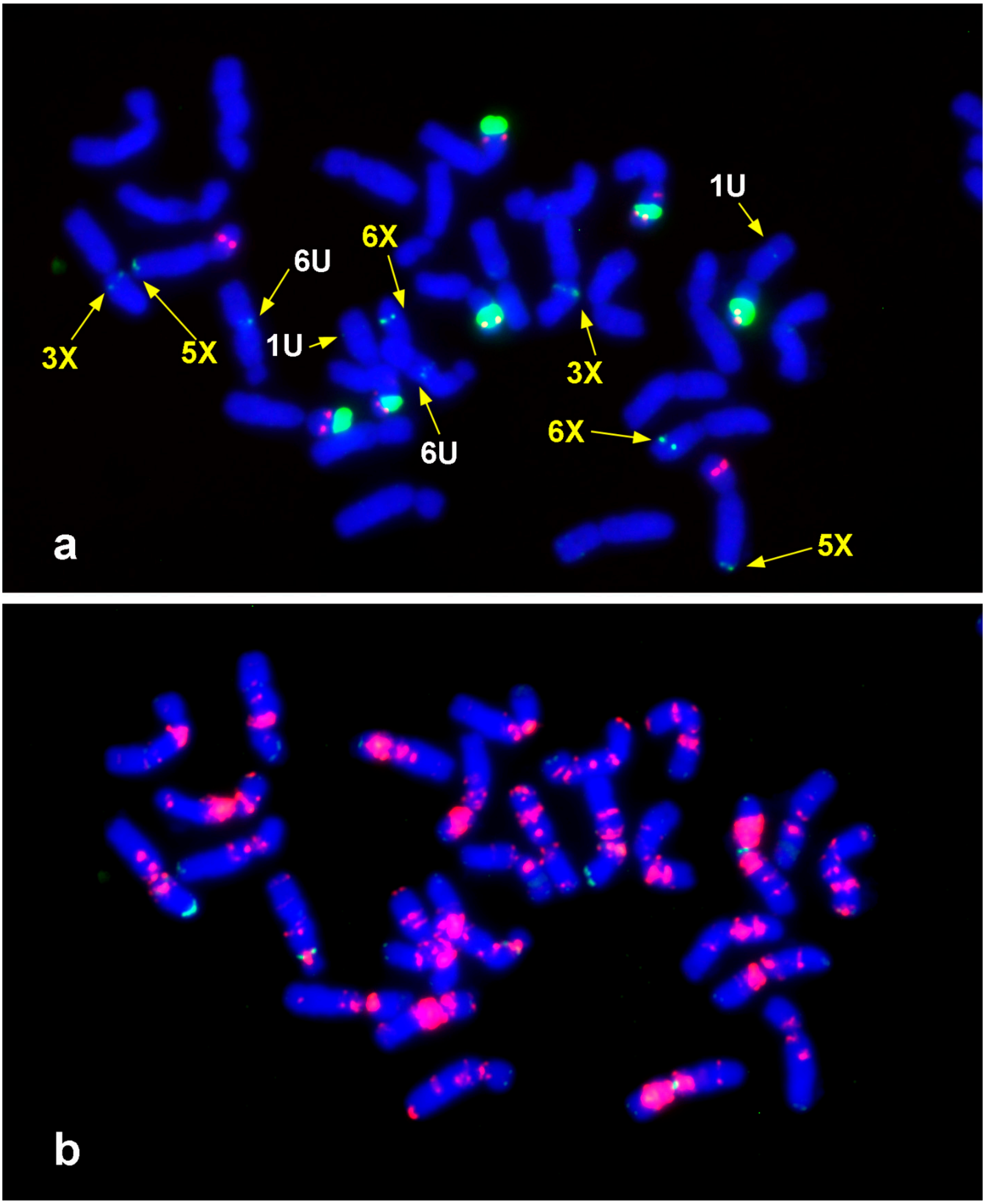

Supplementary Figure S3. Location of minor NORs on chromosomes of *Ae. neglecta,* PI 564182, by sequential FISH with oligo-probes pTa71-1 (green) and pTa794 (red) – a, followed by $(GAA)_n$ (red) and pAs1 (green) - b. Minor NORs are indicated with arrows (a), and the respective chromosomes are identified according to their $(GAA)_n$-FISH patterns (b).

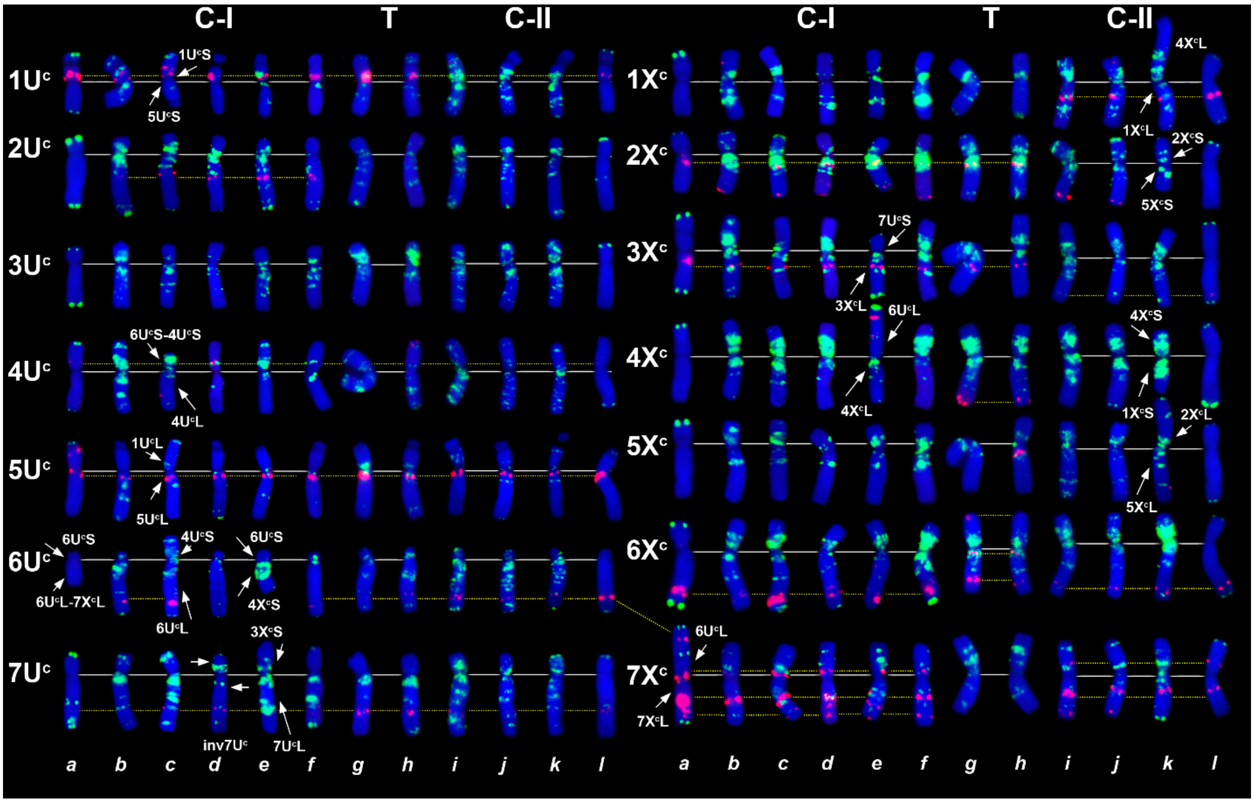

Supplementary Figure S4. Diversity of the pTa-713 probe patterns on chromosomes of *Ae. columnaris,* C-I (*a-f*) in comparison with *Ae. neglecta,* T (*g, h*) and *Ae. columnaris,* C-II (*i-l*): *a* – AE 1607; *b* – K-4241; *c* – K-1193; *d* - i-570045); *e* – PI 486281; *f* – PI 554178; *g* – PI 564182; *h* – IG 170209; *i* – PI 542191; *j* – PI 564181; *k* – TA2084; *l* – PI 564180. Chromosomes are classified based on pSc119.2 (*a, l*) or $(GAA)_n$-labelling patterns (*b – k*), green signals. Hybridization sites of pTa-713 probe are visualized in red. Chromosomes are classified according to genetic nomenclature; translocated chromosomes are designated. Invariable pTa-713 sites are underlined with yellow dotted lines.

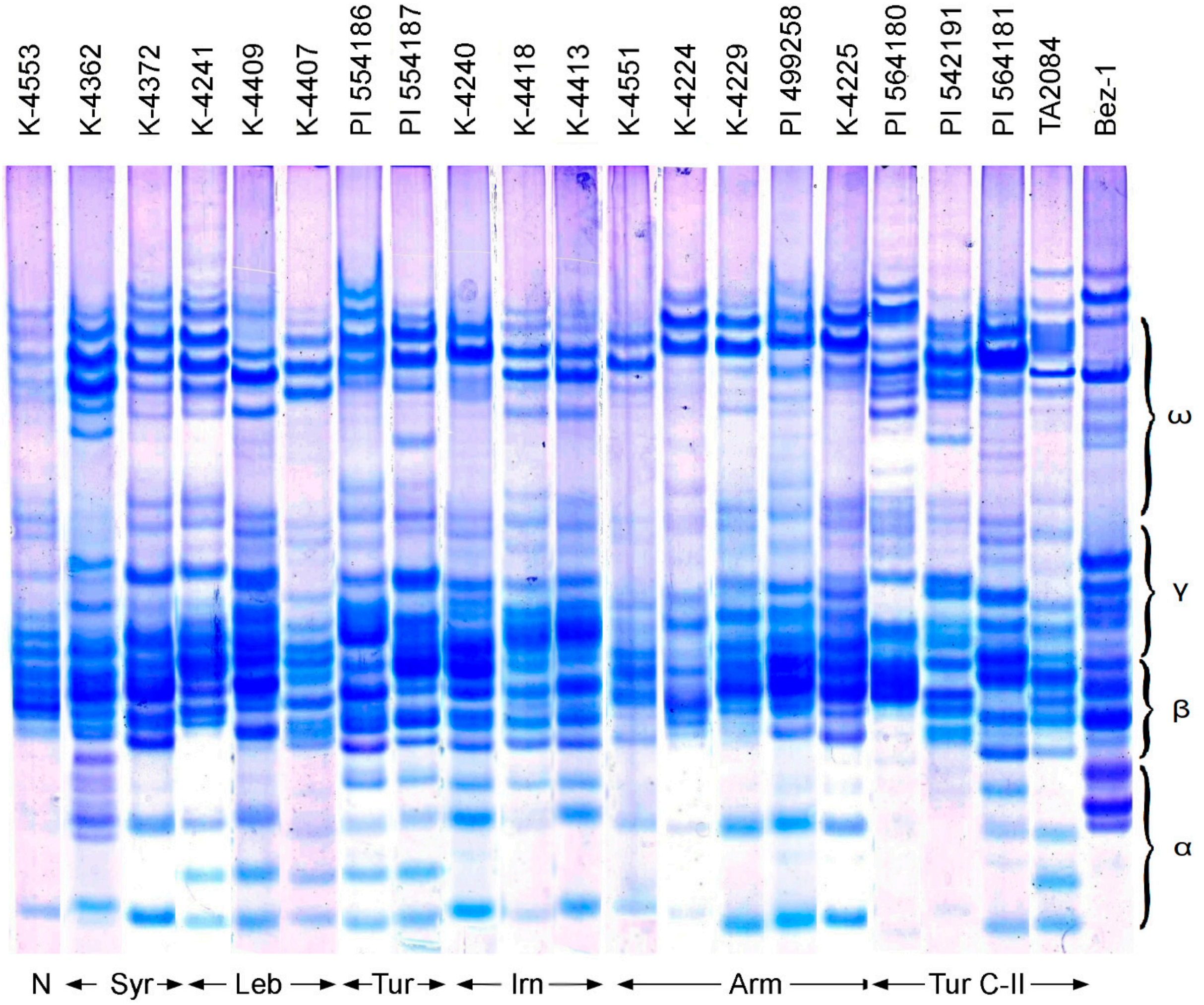

Supplementary Figure S5. Diversity of gliadin spectra of *Ae. columnaris* accessions collected from different countries in comparison with *Ae. neglecta* (N). Accession numbers are shown on the top, while their geographic origin – on the bottom of the figure. α, β, γ, and ω - zones in electrophoretic spectra.

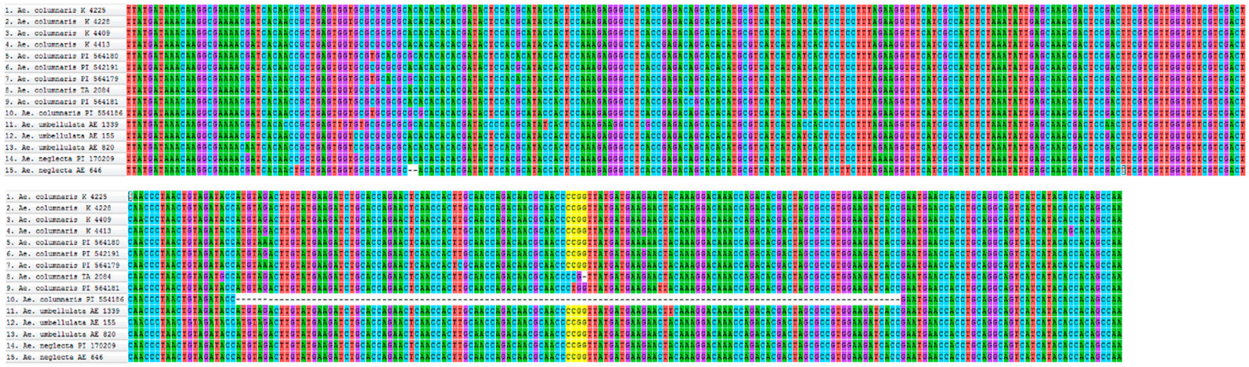

Supplementary Figure S6. Nucleotide variability in the U31 region of analyzed *Aegilops* accessions. The *Msp*I restriction site is highlighted in yellow.

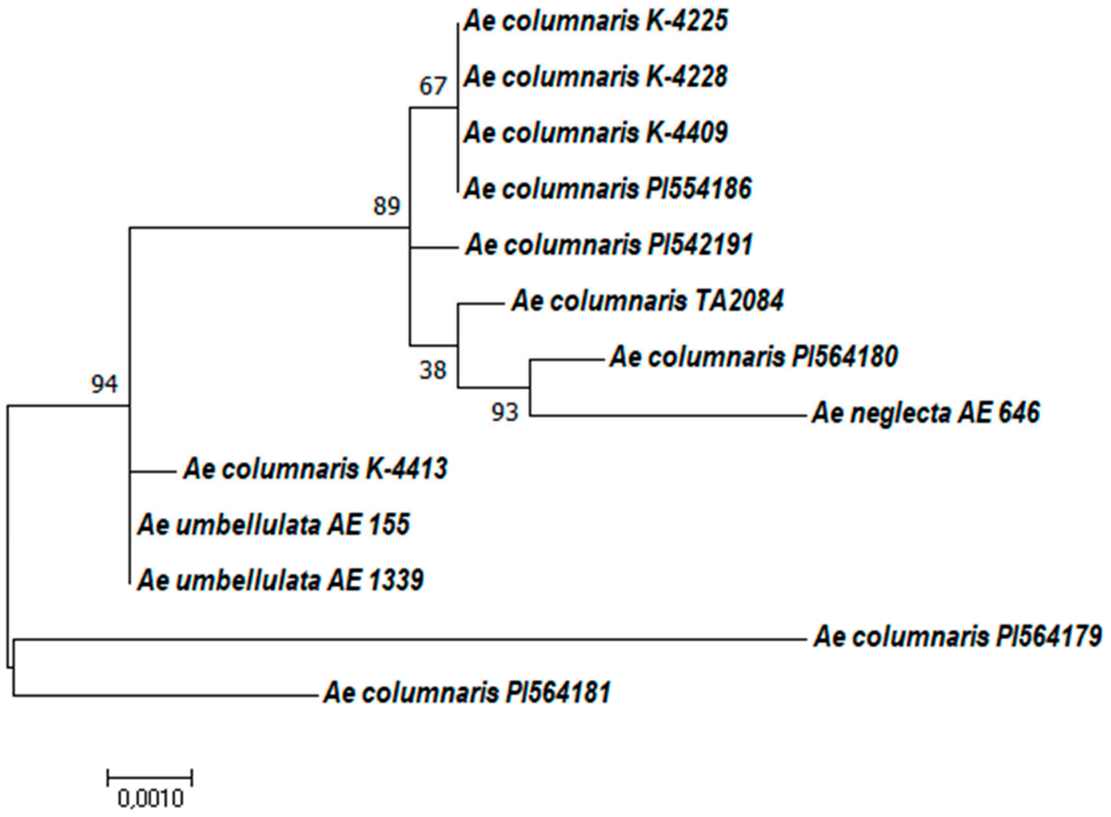

Supplementary Figure S7. Maximum-Likelihood (Tamura-3 parameter model) phylogenetic tree of combined *trnH-psbA, trnT-trnL, rpL32-trnL* sequences; the numbers above the branches indicate bootstrap values: branch length was measured in a number of substitutions per site.
